# Targeting cancer and SARS-CoV-2: phytochemical, biological and molecular dynamic investigations of *Sargassum aquifolium* and *Galaxaura rugosa*

**DOI:** 10.1038/s41598-025-22987-z

**Published:** 2025-11-11

**Authors:** Asmaa S. Abd Elsamiae, Abdel Nasser B. Singab, Ataa Said, Omayma A. Eldahshan, Nada M. Mostafa, Mohamed S. Elnaggar, Asmaa F. Aboul Naser, Abo El-Khair B. El-Sayed, Ahmed A. El-Rashedy, Omnia M. Kutkat, Amal M. El-Feky

**Affiliations:** 1 Pharmacognosy Department, National Research Center, 33 El Bohouth St. (Former El Tahrir St.), Dokki, P.O. 12622, Giza, Egypt; 2 Department of pharmacognosy, Faculty of pharmacy, Ain Shams University, Cairo, 11566 Egypt; 3 Department of Therapeutic Chemistry, National Research Center, 33 El Bohouth St. (Former El Tahrir St.), Dokki, P.O. 12622, Giza, Egypt; 4 Algal Biotechnology Unit, Biological and Agricultural Research Division, National Research Centre, Dokki, Giza, Egypt; 5 Natural and Microbial Products Department, National Research Center, 33 El Bohouth St. (Former El Tahrir St.), Dokki, P.O. 12622, Giza, Egypt; 6 Department of Water Pollution Research, Center of Scientific Excellence for Influenza Virus, National Research Centre, Dokki, Giza, Egypt

**Keywords:** *Sargassum aquifolium*, Galaxaura rugosa, Antioxidant, Antiviral, Anticancer, Biochemistry, Cancer, Chemical biology, Chemistry, Drug discovery, Plant sciences

## Abstract

This study aimed to acquire in-depth phytochemical and biological insights into the different extracts of *Sargassum aquifolium* and *Galaxaura rugosa*, due to the limited existing data on these species. GC/MS analysis of the unsaponifiable matter identified 27 compounds in *S. aquifolium*, including three phytosterols and two fatty alcohols, while *G. rugosa* contained 23 compounds. Fatty acid methyl ester profiling revealed six fatty acids in *S. aquifolium* and 21 in *G. rugosa*. Isolation led to the identification of six compounds from *S. aquifolium* (including two sterols and two fatty alcohols) and seven from *G. rugosa* (with two sterols). GLC analysis showed rhamnose and galactose as the major sugars in *S. aquifolium*, and mannose, arabinose, and glucose in *G. rugosa*. HPLC detected 17 amino acids across both species. LC/MS profiling of pigment extracts revealed 13 pigments, 11 sterols, and 17 terpenoids in *S. aquifolium*, and 16 pigments, 9 sterols, and 12 terpenoids in *G. rugosa*. Among the various extracts, the pet. ether extract of *S. aquifolium* exhibited the highest antioxidant activity, whereas that of *G. rugosa* demonstrated potent antiviral activity against SARS-CoV-2 (IC₅₀ = 6.454 µg/mL). Notably, the aqueous and pigment extracts of *S. aquifolium* showed significant cytotoxicity against lung cancer cells (inhibition rates of 70% and 62.56%, respectively). Furthermore, aqueous extracts of *S. aquifolium* and *G. rugosa* exhibited strong activity against colon cancer cells (76.02% and 61.17% inhibition, respectively). Molecular docking studies highlighted phytol as a potential bioactive compound contributing to these therapeutic effects. These findings suggest that pet. ether extracts could be promising sources of antioxidant and antiviral agents, while the aqueous and pigment extracts of *S. aquifolium* may hold therapeutic potential against lung and colon cancers.

**Supplementary Information:**

The online version contains supplementary material available at 10.1038/s41598-025-22987-z.

## Introduction

In recent years, the global demand for natural resources has markedly increased^1^. Among these, plants are considered a vital source of bioactive compounds with well-established medicinal and nutritional properties^2,3^. Covering approximately 70% of the Earth’s surface, oceans harbor a vast diversity of marine organisms. Algae, in particular, are recognized for their rich repertoire of structurally diverse bioactive metabolites^4^. In several Asian cultures, algae have been traditionally consumed for their health benefits^5^.

Marine algae are known to contain compounds such as fatty acids, steroids, terpenoids, carotenoids, and flavonoids, many of which exhibit a broad spectrum of biological activities, including antioxidant, anticancer, anti-aging, anti-inflammatory, antimicrobial, anticoagulant, antidiabetic, and neuroprotective effects^6–8^.

*Sargassum* species, belonging to the brown macroalgae group, are commonly found in tropical and temperate shallow waters^9^. They are widely distributed across tropical and temperate seas, particularly in the Indo-Pacific region, with *S. aquifolium* commonly occurring along the Red Sea and Indo-Pacific coasts^10^. Their characteristic brown color results from the presence of fucoxanthin, and their cell walls are composed mainly of alginates, laminarins, fucans, and cellulose. Several species of *Sargassum* have demonstrated potent antioxidant and anticancer activities in both in vitro and in vivo studies. Notably, the hexane extract of *S. swartzii* showed cytotoxic effects against T47D and Caco-2 cell lines, likely due to the presence of meroterpenoids^4^.

Furthermore, pet. ether extracts of *S. aquifolium* have been reported to contain bioactive lipids and sterols, including hexadecanoic acid, octadecenoic acid, stigmasterol, and tetradecanoic acid^10^. In addition, phenolic constituents from this species have shown significant antibacterial effects against *Candida albicans*, *Pseudomonas aeruginosa*, and *Escherichia coli*^11^. *Sargassum aquifolium* is also rich in essential amino acids important for human health^12^.

Red macroalgae, on the other hand, owe their coloration to phycoerythrin, one of several accessory photosynthetic pigments such as chlorophylls^13^. They are distributed in tropical and subtropical marine environments worldwide. *Galaxaura rugosa* in particular is found in the Red Sea, Indian Ocean, and Western Pacific, typically inhabiting coral reef ecosystems^14^. These algae hold great potential across industrial, medical, and nutritional sectors due to their antimicrobial, antiviral, antioxidant, anticancer, anti-inflammatory, and antidiabetic properties^15,16^. Studies have shown that *Galaxaura rugosa* is particularly rich in xylose among its monosaccharide components^14^, but comprehensive analyses of its full phytochemical composition remain limited.

From a biomedical perspective, oxidative stress, arising from an imbalance between reactive oxygen species (ROS) and antioxidant defenses, is implicated in the pathogenesis of various diseases, including cancer, neurodegenerative disorders, and chronic inflammation^17^. Marine algae, particularly *Sargassum* species, are known to contain natural antioxidants that can counteract oxidative stress^4^. A study by Nunes et al.^18^ assessed antioxidant-related constituents such as chlorophyll, carotenoids, phenolics, and flavonols in *G. rugosa*, but did not provide a full phytochemical profile. Moreover, lipoidal extracts from *G. rugosa* have shown enhanced anticancer effects, in some cases outperforming vinblastine sulfate^1^. Sulfated polysaccharides from *Sargassum patens* also demonstrated antiviral activity by inhibiting Herpes virus replication^19^, and silver nanoparticles derived from *G. rugosa* have been reported to act against multidrug-resistant bacteria^1^.

Despite these promising findings, there remains a significant gap in the literature regarding the full phytochemical composition and comparative biological properties of different extracts from *S. aquifolium* and *G. rugosa*. This study aims to conduct a comprehensive investigation of these two macroalgae, focusing on their phytochemical profiles and biological activities. The ultimate goal is to identify safe, effective, and naturally derived compounds with potential nutraceutical and therapeutic applications, providing sustainable alternatives to synthetic pharmaceuticals.

## Materials and methods

### Chemicals, reagents, and cell lines

The chemicals and reagents utilized in the research study were of fine analytical grade and were procured from Sigma Co. (USA). Ascorbic acid, doxorubicin and remdesivir were supplied by Pfizer Co., Egypt. DMEM medium (Dulbecco΄s Modified Eagle Medium, Gibco- BRL), MTT salt (Biobasic INC) and dimethyl sulfoxide (DMSO, Merck). Cell lines: MCF-7 [Human Caucasian breast adenocarcinoma], A549 [lung carcinoma] and HCT116 [colon carcinoma] were brought from Karolinska University, Sweden. Cells: 2.4 × 10^4^ VERO-E6 cells (obtained from the Holding Company for Biological Products & Vaccines VACSERA, Egypt). Virus: hCoV-19/Egypt/NRC-03/2020 (Accession Number on GSAID: EPI_ISL_430820).

### Algae material

The harvesting of *S. aquifolium* and *G. rugosa* took place in December 2020, at Hurghada, Red Sea (27°17′N, 33°46′E). The entire thallus was collected, and underwent a drying process at approximately 18 °C for seven days in the shade until complete dryness. Subsequently, the entire algae were ground, and the resulting powders were kept in sealed containers. The identification has been conducted by Dr. El Sayed Hamed and Dr. Mohammed Ezz El-Arab at the National Institute of Oceanography & Fisheries. Voucher specimens numbered PHG-A-SA-414 and PHG-A-GR-415 have been assigned to *S. aquifolium* and *G. rugosa*, respectively.

### Extraction

A total of 750 g of dried powdered *S. aquifolium* and *G. rugosa* was subjected to successive extraction with pet. ether (60–80 °C) at room temperature. The pet. ether extracts were filtered and concentrated under reduced pressure to yield the lipophilic fractions. The remaining defatted residues were then macerated with cold distilled water (3 L × 7 times) at room temperature. The aqueous layers were pooled and evaporated to dryness to obtain the aqueous extracts. For pigment extraction, 250 g of dried powdered material from each alga was extracted using a mixture of pet. ether and acetone (1:1, v/v) at room temperature. The combined filtrates were concentrated at 40 °C using a rotary evaporator under vacuum (Heidolph Laborota 4000, Schwabach, Germany). All pigment extraction steps were conducted in the dark to prevent carotenoid photo-isomerization and photodegradation^20^. The obtained pet. ether, aqueous, and pigment extracts from both macroalgae were stored at 4 °C for subsequent phytochemical and biological analyses.

### Phytochemical investigation of petroleum ether extracts

#### Identification and isolation of lipoidal constituents

Three grams of the pet. ether extracts from *S. aquifolium* and *G. rugosa* were subjected to saponification using alcoholic potassium hydroxide to obtain the unsaponifiable matter (USM). The remaining saponifiable fraction was methylated using boron trifluoride (BF₃) in methanol (14% solution). The fraction was refluxed with 5 mL of BF₃–MeOH reagent for 30 min at 80 °C, then cooled and extracted with n-hexane to yield fatty acid methyl esters (FAME), following the procedures of Tsuda et al.^21^ and Finar^22^. Both USM and FAME were analyzed by gas chromatography–mass spectrometry (GC/MS) for the identification of sterols, hydrocarbons, fatty acids, and other lipophilic constituents, as described by Hussiny et al.^23^ and Rabie et al.^24^. The USM fractions were further separated by preparative thin-layer chromatography (TLC) on silica gel F₂₅₄-coated plates (20 × 20 cm) using benzene: ethyl acetate (8:2, v/v) as the mobile phase. The bands were visualized with a 10% sulfuric acid spray reagent. Individual bands were collected, and further purification was performed using TLC with a chloroform: methanol (9:1, v/v) solvent system^25^. This process yielded six compounds from *S. aquifolium* and seven compounds from *G. rugosa*. The structures of the purified compounds were elucidated using spectroscopic techniques;

Fourier-transform infrared (FT-IR) spectra of the purified compounds were recorded using a Shimadzu FTIR spectrophotometer in the range of 4000–400 cm^–1^ (KBr discs). Nuclear magnetic resonance (NMR) spectra, including ¹H-NMR and ¹³C-NMR, were obtained on a Bruker Avance 400 MHz spectrometer, with chemical shifts (δ) expressed in parts per million (ppm) relative to tetramethylsilane (TMS) as an internal standard, using CDCl₃ as solvent. Mass spectrometric data (MS) were recorded on an Agilent 5977B GC/MSD system equipped with an EI source at 70 eV.

#### Phytochemical investigation of aqueous extracts

The total polysaccharide content in *S. aquifolium* and *G. rugosa* was quantified using the phenol–sulfuric acid method. Polysaccharides were subsequently precipitated by the addition of absolute ethanol, following the procedure of Singab et al.^26^. Identification of the isolated polysaccharides was carried out using gas–liquid chromatography (GLC), as described by El-Feky et al.^27^. In parallel, the total protein content was determined by the micro-Kjeldahl method. Proteins were precipitated using trichloroacetic acid according to the method of Ibrahim et al.^28^. The precipitated proteins were then hydrolyzed and analyzed for amino acid composition using high-performance liquid chromatography (HPLC) with the Pico-Tag method, following the protocol established by the Millipore Cooperative^29^.

#### Phytochemical investigation of pigment extracts

The contents of carotenoids, total chlorophylls, and chlorophylls *a* and *b* were quantified using spectrophotometric methods, with absorbance measured at 470, 651, and 664 nm. Concentrations were calculated using the equations described by El-Feky et al.^30^. For the qualitative identification of phytoconstituents, an XEVO TQD triple-quadrupole mass spectrometer was used in both positive and negative electrospray ionization (ESI) modes, and data were processed with MassLynx 4.1 software (Waters Corporation, Milford, MA, USA). The HPLC–MS system was equipped with an autosampler (Switzerland) and fitted with a UPLC-BEH C18 ACQUITY column (1.7 μm, 2.1 × 50 mm). The mobile phase consisted of two eluents: (A) water with 0.1% formic acid and (B) methanol with 0.1% formic acid. A gradient elution program was applied as follows: 30% B at 0–2 min, linearly increased to 100% B over 15 min, held at 100% B for 5 min, then returned to initial conditions within 3 min, for a total run time of 25 min, at a flow rate of 0.2 mL/min, following the method of El Sawi et al.^31^,.

#### Biological investigations of the macroalgal extracts

Several biological studies have been undertaken focusing on pet. ether, aqueous, and pigment extracts from *S. aquifolium* and *G. rugosa*.

#### Free radical scavenging activity

The antioxidant activity of pet. ether, aqueous, and pigment extracts from *S. aquifolium* and *G. rugosa* were assessed in vitro using the DPPH free-radical scavenging method as described by Singab et al.^26^. This evaluation was conducted at concentrations of 10 µg and 50 µg for each extract, with ascorbic acid serving as the reference standard^32^.

#### Antiviral activity

The antiviral activity of the tested extracts against SARS-CoV-2 (strain hCoV-19/Egypt/NRC-03/2020, Accession Number on GISAID: EPI_ISL_430820) was assessed using the MTT assay, following the protocol of Hemdan et al.^33^, with remdesivir serving as the positive control. VERO-E6 cells were cultured in Dulbecco’s Modified Eagle’s Medium (DMEM) supplemented with 10% heat-inactivated fetal bovine serum (FBS), 100 U/mL penicillin, and 100 µg/mL streptomycin, and maintained at 37 °C in a humidified incubator containing 5% CO₂. Viral stocks were propagated in VERO-E6 cells and titrated using the TCID₅₀ method. To determine the maximum non-toxic concentration (MNTC), extracts from *S. aquifolium* and *G. rugosa* were dissolved in 10% DMSO in distilled deionized water and serially diluted in DMEM. VERO-E6 cells were seeded in 96-well plates (2 × 10⁴ cells/well) and incubated for 24 h before treatment. The cells were exposed to different extract concentrations for 72 h, after which MTT solution (20 µL of 5 mg/mL) was added to each well. Following 4 h of incubation, the resulting formazan crystals were solubilized in DMSO, and absorbance was measured at 570 nm using an Anthos Zenyth 200RT plate reader (Anthos Labtec Instruments, Heerhugowaard, Netherlands). The CC₅₀ (concentration that reduces cell viability by 50%) was then calculated. For antiviral evaluation, VERO-E6 monolayers were seeded in 96-well plates and allowed to adhere overnight. The cells were infected with SARS-CoV-2 (MOI = 0.1) for 1 h at room temperature to allow viral adsorption, followed by removal of the viral inoculum. Thereafter, the cells were treated with DMEM containing serial concentrations of the extracts and incubated for 72 h. The MTT assay was performed as described above, and viral inhibition was expressed as a percentage relative to untreated controls. The IC₅₀ (concentration required to inhibit 50% of viral replication) was determined from the dose–response curve.

#### Anticancer activity

The cytotoxic effects of the petroleum ether, aqueous, and pigment extracts of *S. aquifolium* and *G. rugosa* were evaluated against three human cancer cell lines: MCF-7 (breast adenocarcinoma), A549 (lung carcinoma), and HCT-116 (colon carcinoma). Doxorubicin was used as the reference standard drug^34^. The assay was performed using the MTT method as originally described by Mosmann^35^ and later adapted by El-Feky and Mohammed^36^. All cell lines were obtained from the American Type Culture Collection (ATCC, Rockville, MD, USA) and cultured in RPMI-1640 medium supplemented with 10% fetal bovine serum (FBS), 100 U/mL penicillin, and 100 µg/mL streptomycin. The cells were maintained at 37 °C in a humidified atmosphere containing 5% CO₂. For the cytotoxicity assay, cells were seeded in 96-well plates at a density of 1 × 10⁴ cells/well and incubated for 24 h to allow attachment. The extracts were dissolved in 10% DMSO and serially diluted to obtain a concentration range of 1–100 µg/mL. Cells were treated with these concentrations for 48 h under standard culture conditions. Following treatment, 20 µL of MTT solution (5 mg/mL) was added to each well, and the plates were incubated for an additional 4 h. The culture medium was then carefully removed, and the resulting formazan crystals were solubilized in 150 µL of DMSO. Absorbance was measured at 570 nm using an Anthos Zenyth 200RT plate reader (Anthos Labtec Instruments, Heerhugowaard, Netherlands). Cell viability was expressed as a percentage of the untreated control, and the IC₅₀ values (concentrations reducing cell viability by 50%) were calculated by plotting dose–response curves using GraphPad Prism software.

### Statistical analysis and calculations

Statistical analysis was conducted using one-way analysis of variance (ANOVA), followed by post hoc comparisons where applicable to assess differences between groups. The analysis was performed using CoStat software (CoHort Software, Monterey, CA, USA). A *p*-value of less than 0.05 was considered statistically significant.

### Computer-guided docking study of the isolated compounds

#### System preparation and molecular docking

The three-dimensional structures of the main protease (Mpro) of SARS-CoV-2 (PDB ID: 6LU7) as reported by Jin et al.^37^ and the Vascular Endothelial Growth Factor Receptor 2 (VEGFR2) (PDB ID: 4ASD) as detailed by McTigue et al.^38^ were sourced from the Protein Data Bank^39^ and were prepared utilizing UCSF Chimera^40^. The pH was set and optimized to 7.5 using PROPKA^41^. The two-dimensional structure was generated with ChemBioDraw Ultra 12.1^42^. For energy minimization, the steepest descent method and the MMFF94 force field were employed within Avogadro software^43^ to optimize the 2D structure. Before docking, hydrogen atoms were eliminated using UCSF Chimera^40^.

#### Molecular docking

AutoDock Vina was employed for the docking calculations^44^, with Gasteiger partial charges^45^ assigned during the docking process. The AutoDock graphical user interface provided by MGL tools was utilized to define the AutoDock atom types^46^. The coordinates for the AutoDock Vina grid center were set at −26.7633, −7.98758, −9.31384 for the 6LU7 structure and − 8.23794, 13.5703, 72.3436 for the 4ASD structure. The dimensions of the search space were established at 20 Å x 20 Å x 20 Å for both cases, with an exhaustiveness parameter of 8. The Lamarckian genetic algorithm^47^ was employed to generate docked conformations ranked in descending order according to their docking energy^16^.

#### Molecular dynamics (MD) simulations

Molecular Dynamics (MD) simulations were conducted utilizing the GPU version of the PMEMD engine included in the AMBER 18 software package^48^. The partial atomic charge for each compound was determined using the General Amber Force Field (GAFF) method provided by ANTECHAMBER^49^. The Leap module from the AMBER 18 software package was employed to implicitly solvate each system within an orthorhombic box containing TIP3P water molecules and facilitate the neutralization of each system by adding Na + and Cl- counter ions. To achieve an isobaric-isothermal (NPT) ensemble, the number of atoms and pressure were held constant throughout each production simulation, with the pressure regulated at 1 bar using the Berendsen barostat^50^. The SHAKE algorithm was employed. A step size of 2 femtoseconds was utilized, integrating an SPFP precision model. The simulations were conducted under an isobaric-isothermal ensemble (NPT) framework, featuring randomized seeding, a constant pressure of 1 bar, a pressure-coupling constant of 2 picoseconds, a temperature of 300 K, and a Langevin thermostat with a collision frequency of 1 picosecond.

#### Post-MD analysis

The trajectories generated from molecular dynamics simulations, saved at intervals of 1 ps, were subsequently analyzed utilizing the CPPTRAJ module from the AMBER18 suite^51^. For the creation of all graphs and visualizations, the Origin data analysis program^52^ and Chimera software^40^ were employed.

#### Thermodynamic calculation

The Poisson-Boltzmann or generalized Born and surface area continuum solvation methods, specifically MM/PBSA and MM/GBSA, have proven effective in estimating ligand-binding affinities^53^. The molecular simulations of protein-ligand complexes conducted using MM/GBSA and MM/PBSA calculate the binding free energy through a rigorous statistical-mechanical approach within a specified force field. The binding free energy is averaged over 2000 snapshots taken from a comprehensive 200 ns trajectory. The change in binding free energy (ΔG) for each molecular entity, including the complex, ligand, and receptor, can be expressed according to Hou et al.^54^.

**Ethical approval. **The study was compiled with the ethical guidelines set by the Medical Ethical Committee of the National Research Centre, Egypt (Approval No.04210521).

## Results

### Phytochemical investigation of the petroleum ether extracts

#### Identification of lipoidal constituents

The identification of compounds in the unsaponifiable matter (USM) and fatty acid methyl esters (FAME) of *S. aquifolium* and *G. rugosa* was performed using gas chromatography–mass spectrometry (GC/MS). Retention times, retention indices, and mass spectral fragmentation patterns were compared with those in established database libraries for compound identification. Quantitative analysis was carried out based on the relative peak areas^55^. The detailed GC/MS data, including identified compounds, retention times, and spectral interpretations, are presented in Supplementary Tables 1 and 2. Corresponding GC/MS chromatograms for the USM and FAME of both species are shown in Supplementary Figs. 1 and 2.

The USM of *S. aquifolium* was found to contain 27 compounds, including 19 hydrocarbons, 3 sterols (fucosterol, cholesterol, and α-androstan-17-one—the latter reported here for the first time), 2 alkenes, 2 fatty alcohols (1-heptacosanol and phytol—both newly identified in this species), and 1 glucoside. In contrast, the USM of *G. rugosa* comprised 23 compounds: 19 hydrocarbons, 1 sterol (cholesterol), and 3 alkenes. Notably, both species shared methyldodecylbenzene and methylundecylbenzene as major hydrocarbon components. However, the most abundant hydrocarbon in *S. aquifolium* was ethyldecylbenzene, while ethylundecylbenzene predominated in *G. rugosa*.

GC/MS analysis of the fatty acid methyl ester (FAME) fractions revealed six fatty acids in *S. aquifolium*, including hexadecanoic acid and 10-octadecenoic acid, both of which are reported for the first time in this species. In contrast, *G. rugosa* was found to contain 21 distinct fatty acids. Among them, 3-octyloxiraneoctanoic acid and 13-docosenoic acid are newly identified in this species in the current study.

#### Structure elucidation of the isolated compounds

A total of six compounds were isolated from *S. aquifolium*, which included two sterols: desmosterol and hydroxycholestan-5-yl acetate, two alkenes: tetracosene and nonadecene, and two fatty alcohols: nonadecanol and phytol. While, seven compounds were obtained from *G. rugosa*, comprising two sterols: cholesterol and campsterol, and five hydrocarbons, specifically 7-phenyl eicosane, 2-phenyl tridecane, 4-phenyl dodecane, 6-phenyl dodecane, and 2-phenyl undecane.

**Compound 1**: From *S. aquifolium* as a solid, R*f* of 0.39 in a benzene-ethyl acetate (8:2 v/v), with a melting point of 122 °C. The mass spectrum revealed a molecular weight peak at *m/z* 384 (87%), corresponding to the molecular formula C_27_H_44_O. Additionally, several significant peaks were observed: *m/z* 368 (25%) attributed to the loss of a methyl group, *m/z* 365 (20%) due to the loss of water, *m/z* 350 (22%) resulting from the simultaneous loss of a methyl group and water, and *m/z* 298 (50%), *m/z* 270 (80%), *m/z* 254 (100%) associated with the loss of the alkyl side chain and water. Other notable peaks included m/z 252 (30%), *m/z* 228 (25%), and *m/z* 212 (85%). These findings corroborated the identification of desmosterol^56^.

**Compound 2**: From *S. aquifolium* as a white solid, with an R*f* of 0.34 in the benzene-ethyl acetate (8:2 v/v). MS *m/z* represents a molecular weight of 446 with a molecular formula of C_29_H_50_O_2_. Other main fragments were 428(40%) due to loss of water, 368(30%), 43(10%), 41(10%) and 55(20%). It was concluded that the isolated compound is hydroxycholestan-5-yl acetate as reported by You and Wang^57^.

**Compound 3**: From *S. aquifolium* as a liquid with R*f* of 0.26 in the benzene-ethyl acetate (8:2 v/v). MS *m/z* represents a molecular weight of 336, corresponding to the molecular formula C_24_H_48_. One major fragment peak appeared at *m/z* 168(100%). Upon reconstruction of two fragments, each equivalent to *m/z* 168. The existence of a single fragment is due to the symmetric nature of this compound. These findings corroborate the identification of tetracosene, as documented by Gay^58^.

**Compound 4**: From *S. aquifolium* as a colorless solid with R*f* of 0.65 in benzene-ethyl acetate (8:2 v/v) and melting point 230 °C. FT-IR (λmax) 2960, 2925, and 2855 cm^−1^. MS *m/z* represents a molecular weight of 266 with a molecular formula of C_19_H_38_. Other main fragments were 265 (70%), 97(90%), 111(40%), 83(20%) and 43(10%). It was deduced that the isolated compound is Nonadecene as reported by Amudha et al.^59^.

**Compound 5**: From *S. aquifolium* as a colorless needle crystal exhibiting R*f* of 0.18 in benzene-ethyl acetate (8:2v/v), and a melting point of 64 °C. The infrared spectrum displayed a distinctive absorption band at 3425 cm^−1^, indicative of O-H stretching. Absorption peaks at 2935 and 2866 cm^−1^ correspond to aliphatic C-H stretching, while weaker frequencies at 1458 cm^−1^ and 1375 cm^−1^ are attributed to C-H bending. The ^1^H-NMR spectrum (CDCl_3_, 400 MHz, δ ppm) revealed signals at 2.69 (t, J = 6.9 Hz, H-1), 2.37 (s, O-H), 1.98 (m, H-2), 1.32 (m, H-3 to H-18), and 0.95 (t, J = 7.4 Hz, H-19). The ^13^C NMR spectrum (CDCl_3_, 100 MHz, δ ppm) showed signals at 59.4 (C-1), 34.9 (C-2), 32.5 (C-3), 29.91–29.31 (C-4 to C-14), 27.5 (C-15), 26.3 (C-16), 24.1 (C-17), 21.3 (C-18), and 15.6 (C-19). Based on these findings and a comparison with existing literature^60^, it was concluded that compound 5 is nonadecanol.

**Compound 6**: From *S. aquifolium* as solid, with R*f* of 0.68 in the benzene-ethyl acetate (8:2 v/v) and melting point 250 °C. The FT-IR analysis revealed a λmax of 2900 cm^−1^, indicative of alkyl C-H stretching, while the range of 3250 cm^−1^ to 3500 cm^−1^ was associated with broad OH stretching. Additionally, a peak at 1450 cm^−1^ was attributed to the C = C bond (due to α–OH), and a peak at 1005 cm^−1^ corresponded to C-O stretching. The mass spectrometry analysis indicated a molecular weight of 296, corresponding to the molecular formula C_20_H_40_O. Notable fragments included 71(100%), 149 (20%), 123 (10%), 221 (10%), and 179(4%). Based on the data presented by Byju et al.^61^, it was concluded that compound 6 is phytol.

**Compound 7**: From *G. rugosa* as a faintly yellow solid, with R*f* of 0.31 in benzene-ethyl acetate (8:2 v/v) and melting point 149 °C. FT-IR (λmax) 3437, 2933, 1631, 1467, 1381, 1056, 958, 842 cm^−1^. ^1^H-NMR(CDCl_3_, 400 MHz, δ ppm): δ 5.98 (1H, br d, *J* = 5.6 Hz, H-6), 3.9 (1H, br dd, *J* = 6.7 Hz, H-3), 2.46 (1H, d, *J* = 6.7 Hz, H-4), 1.48 (3 H, s, H-19), 0.99 (1H, d, *J* = 6.9 Hz, H-21), 0.85 (3 H, d, *J* = 7.0 Hz, H-27), 0.70 (3 H, d, *J* = 7.0 Hz, H-26), 0.62 (3 H, s,H-18);^13^C-NMR (CDCl3, 100 MHz, δ ppm): δ150.4 (C-1), 123.8(C-2), 76.3(C-3), 58.2(C-4), 56.1(C-5), 50.6(C-6), 43.7(C-7), 40.2(C-9), 38.1(C-10), 37.5(C-11), 36.1(C-12), 35.0(C-13), 33.9(C-14), 32.5 (C-15), 30.1(C-17), 28.0(C-18), 26.3(C-19), 22.3(C-20), 21.4(C-21), 20.0(C-22), 19.3(C-23), 18.1(C-24), 16.7(C-25), 12.4(C-26); MS *m/z* represent a molecular weight of 386(100%) with a molecular formula of C_27_H_46_O. Other main fragments were 368 (70%), indicating the loss of water, 107 (90%), 145(85%), 213 (80%), and 275 (100%). The above results confirmed the identity of cholesterol as reported in Zipser et al.^62^.

**Compound 8**: From *G. rugosa* as white amorphous powder with R*f* of 0.56 in benzene-ethyl acetate (8:2 v/v) and melting point 158 °C. FT-IR (λmax) 3400, 1640,1050, 845, 830, 802 cm^−1^; ^1^H-NMR (CDCl_3_, 400 MHz, δ ppm): 5.12 (1H, br d, *J* = 4.9 Hz, H-6), 3.46 (1H, br dd, *J* = 7.0 Hz, H-3), 2.1 (1H, d, *J* = 7.0 Hz, H-4), 1.42 (3 H, s, H-19), 0.87 (1H, d, *J* = 6.9 Hz, H-21), 0.75 (3 H, d, *J* = 7.1 Hz, H-27), 0.63 (3 H, d, *J* = 7.0 Hz, H-26), 0.58 (3 H, d, *J* = 7.0 Hz, H-28), 0.50 (3 H, s,H-18); 13 C-NMR (CDCl_3_, 100 MHz, δ ppm): δ143.5 (C-5),125.3 (C-6), 74.9 (C-3), 57.8 (C-14), 56.4 (C-17), 52.5 (C-9), 46.7 (C-24), 42.6 (C-4), 41.2 (C-13), 39.6 (C-12), 38.6(C-1), 37.5 (C-10), 35.2 (C-25), 33.7 (C-22), 32.6 (C-7),31.5 (C-20), 30.4 (C-8), 29.7 (C-2), 27.6 (C-16), 25.6 (C-28),23.2 (C-15), 21.4 (C-23), 20.3 (C-18), 19.7 (C-11), 18.2(C-21), 17.0 (C-27), 16.3 (C-26), 12.5 (C-19); MS *m/z* (rel int. (%): 400 (M.wt) (40), 385 (78), 382 (13), 367 (97),315 (32), 289 (13), 213(100). MS represents the molecular formula C_28_H_48_O. Upon reviewing the data presented, compound 8 has been conclusively identified as 24(R)-methylcholesta-5-en-3β-ol (campsterol) by correlating multiple physical and spectral characteristics with those documented in the literature^63^.

**Compound 9**: From *G. rugosa* as an amorphous solid with R*f* of 0.08 in benzene-ethyl acetate (8:2 v/v). MS *m/z* represents a molecular weight of 358 with a molecular formula of C_26_H_46_. Other main fragments were 91 (100%), 175 (40%), 43 (20%) and 105 (10%). Identification of the compound as 7-phenyl Eicosane has been validated, according to the findings of Youssef et al.^64^.

**Compound 10**: From *G. rugosa* as an amorphous solid with R*f* of 0.20 in the benzene-ethyl acetate (8:2 v/v). MS *m/z* represents a molecular weight of 260 (10%) with a molecular formula of C_19_H_32_. Other main fragments were 183 (40%), 105 (100%) and 91(20%). Consequently, compound 10 has been identified as 2-phenyl tridecane, as documented by El Hawary et al.^65^ and EL-Hefny et al.^66^.

**Compound 11**: From *G. rugosa* as an amorphous solid with R*f* of 0.64 in benzene-ethyl acetate (8:2 v/v). MS *m/z* represents a molecular weight of 246 with a molecular formula of C_18_H_30_. Other main fragments were 246(20), 204(30), 133(60), 105(20) and 91(100). Thus, the isolated compound was found to be 4-phenyl-dodecane as reported by EL-Hefny et al.^66^.

**Compound 12**: From *G. rugosa* as an amorphous solid with R*f* of 0.72 in benzene-ethyl acetate (8:2 v/v). MS *m/z* represents a molecular weight of 246 with a molecular formula of C_18_H_30_. Other main fragments were 246(20), 175(30), 161(40), 119(20), 105(25) and 91(100), Therefore, compound 12 was identified as 6-phenyl-dodecane as reported by EL-Hefny et al.^66^.

**Compound 13**: From *G. rugosa* as an amorphous solid with R*f* of 0.49 in the benzene-ethyl acetate (8:2 v/v). MS *m/z* represents a molecular weight of 232 with a molecular formula of C_17_H_28_. Other main fragments were 232(15), 105(100), and 91(10). Thus, compound 13 has been characterized as 2-phenyl undecane as reported by EL-Hefny et al.^66^.

#### Phytochemical investigation of the isolated polysaccharides

The total carbohydrate content of *S. aquifolium* and *G. rugosa* was determined to be 18.07% and 11.10% w/w, respectively. The monosaccharide composition of the isolated polysaccharides, along with the GLC chromatograms for both macroalgae, is presented in Supplementary Tables 3 and Supplementary Fig. 3. The results indicate that rhamnose 21.75% and galactose 13.68% are the most abundant sugars in *S. aquifolium*, whereas mannose 37.40%, arabinose 22.55%, and glucose 11.44% are the predominant sugars in *G. rugosa*.

#### Phytochemical investigation of the precipitated proteins

The total protein content of *S. aquifolium* and *G. rugosa* was determined using the micro-Kjeldahl method, whereby the nitrogen content was multiplied by a factor of 6.25. The protein contents were calculated as 12.5% and 15% (w/w of the dry powder), respectively. The composition and concentrations of amino acids in the extracted proteins are presented in Supplementary Tables 4 and illustrated in Supplementary Fig. 4. A total of seventeen amino acids were identified in both macroalgae species. In *S. aquifolium*, the predominant amino acids were threonine (136.03 mg/g), aspartic acid (96.48 mg/g), and histidine (93.40 mg/g). In contrast, *G. rugosa* exhibited higher levels of histidine (122.38 mg/g), tyrosine (97.87 mg/g), and aspartic acid (88.21 mg/g). These results suggest that both *S. aquifolium* and *G. rugosa* are rich sources of essential and non-essential amino acids, underscoring their potential nutritional value.

### Phytochemical investigation of macroalgal pigment extract

#### Quantitative estimation of natural pigments

The findings detailed in Supplementary Table 5 revealed that *S. aquifolium* possesses a higher pigment content of chlorophyll and carotenoids than *G. rugosa*. The total chlorophyll concentration in *S. aquifolium* was recorded at 33.46 µg/ml, in contrast to *G. rugosa*, which had a total of 26.11 µg/ml. In terms of carotenoids, *S. aquifolium* exhibited a level of 7.54 µg/ml, while *G. rugosa* showed a significantly lower level of 2.87 µg/ml.

#### Liquid chromatography–tandem mass spectrometry

Positive and negative ionization techniques using LC/ESI-MS were utilized for the pigment extracts of *S. aquifolium* and *G. rugosa*. The resulting chromatograms are illustrated in Supplementary Fig. 5. The identification of potential compounds was performed by analyzing molecular mass, mass fragmentation patterns, and referencing pertinent literature, as detailed in Supplementary Tables 6 and 7.

A total of 41 compounds were identified in *S. aquifolium*, comprising 13 pigments, 11 sterols, and 17 terpenoid compounds. Of these, 18 compounds were observed in the positive ion mode, whereas 6 compounds were detected in the negative ion mode. Notably, 17 compounds were found in both ion modes. Thus, the majority of compounds were identified in the positive ion mode. While the analysis of *G. rugosa* revealed a total of 37 compounds, consisting of 16 pigments, 9 sterols, and 12 terpenoid compounds. Among these, 5 compounds were detected in the positive ion mode, whereas 21 were identified in the negative ion mode. It is noteworthy that 11 compounds were observed in both modes. Consequently, the predominant number of compounds was found in the negative ion mode.

The analysis revealed the presence of 7 pigments in both *S. aquifolium* and *G. rugosa*, specifically cryptoxanthin, canthaxanthin, 19-but-fucoxanthin, beta-carotene, adonirubin, erythrin, and pelargonidin. Additionally, 6 sterols were detected in both macroalgae, which include campestanol, cycloartenol, ergosterol, campesterol, fucosterol, and brassicasterol. Moreover, 8 terpenes were identified as common to both macroalgae, comprising stypotriol triacetate, phytol tuberatolide B, labdane, cyclosadol, prostane, and two farnesylacetone derivatives: (5E,10Z)−6,10,14-trimethyl pentadeca-5,10-dien-2,12-dione and (5E,9E,13E)−6,10,14-trimethyl pentadeca-5,9,13-trien-2,12-dione^67^.

Carotenoids and chlorophyll exemplify important pigments found in nature. The characterization of these carotenoids was accomplished through LC/MS-MS, which confirmed the presence of specific molecular ions, including dehydrated [M + H–H_2_O]^+^ and protonated [M + H]^+^. The results from the mass spectrometry analysis demonstrated that lutein primarily exists in the dehydrated form, corroborating previously published findings^68^. In the case of fucoxanthin, a significant presence of the protonated ion (*m/z* 659) was detected; however, the dehydrated molecule *m/z* 641 was selected to facilitate comparison with the standard. For canthaxanthin, the protonated molecule was recognized as predominant^69^.

Beta-carotene has the chemical formula C_40_H_56_ and a molecular weight of 536. The mass spectrum reveals the deprotonated molecular ion at *m/z* 535, which serves as the base peak. A fragment ion at *m/z* 135, identified as [M-H-400]^-^, is associated with the cleavage between the 7th and 8th carbons of the polyene chain. Furthermore, a fragment ion at *m/z* 444 corresponds to the elimination of toluene, indicated as [M-H-92 + 1]^-^. Another fragment ion at *m/z* 429, noted as [M-H-106]^-^, is related to the loss of xylene. Additionally, the rupture of carbon-carbon double bonds at C11-C12, C13-C14, C15-C16, and C17-C18, along with hydrogen transfer to the ions, generates fragment ions at *m/z* 201, 241, 267, and 293. These observations are consistent with the findings of Ibrahim et al.^70^.

Lutein, a hydroxycarotenoid, possesses a molecular weight of 568 and is represented by the formula C_40_H_56_O_2_. The deprotonated molecular ion was observed at *m/z* 567, which was characterized by its fragmentation pattern. According to the findings of Ibrahim et al.^70^, the fragment ion typically found at *m/z* 549, indicative of a compound with a hydroxyl group, results from the loss of a water molecule [M-H-18]^-^, establishing it as the base peak. Additionally, fragment ions at *m/z* 475 and 428 [M-H-139]^-^ are linked to the removal of toluene [M-H-92]^-^ and the loss of the terminal ring that includes the unconjugated C3-C4 and C7-C8 double bonds, respectively. These fragments are valuable for differentiating lutein from its isomer, zeaxanthin. Moreover, the fragment ion at *m/z* 411 [M-H-138-18]^-^ is associated with the removal of the β-ring containing a hydroxyl group, which occurs through the cleavage of the C23-C24 double bond.

Diatoxanthin, a hydroxycarotenoid, has the chemical formula C_40_H_54_O_2_ and a molecular weight of 566. The fragmentation pattern reveals the protonated molecular ion [M + H]^+^ at *m/z* 567. The loss of one water molecule [M + H-18]^+^ and xylene generates a fragment ion at *m/z* 549, which further produces a molecular ion at *m/z* 443 [M + H-18–106]^+^. Moreover, fragments detected in our analysis, as noted by Ibrahim et al.^70^, include *m/z* values of 443, 217, 199, 310, 175, and 145.

### Biological investigations

#### Free radical scavenging activity

As presented in Supplementary Table 8, the pet. ether extract of *S. aquifolium* exhibited the highest DPPH radical scavenging activity, followed by the pet. ether and aqueous extracts of *G. rugosa*. Moderate antioxidant activity was observed in both the pigment extract of *G. rugosa* and the aqueous extract of *S. aquifolium*. Notably, the pigment extract of *S. aquifolium* demonstrated DPPH inhibition rates of 68.80% and 69.20% at concentrations of 10 and 50 µg/mL, respectively. These results suggest that the extracts of *S. aquifolium* and *G. rugosa* generally exert a concentration-dependent free radical scavenging effect, with the exception of the aqueous extract of *G. rugosa*, which did not follow this trend.

#### In-vitro antiviral activity against SARS-CoV-2

As shown in Supplementary Tables 9 and 10, and in comparison to the reference antiviral drug remdesivir, the pet. ether extract of *G. rugosa* exhibited the highest antiviral activity against SARS-CoV-2, with a CC₅₀ of 6,786.207 µg/mL and an IC₅₀ of 6.454 µg/mL. This was followed by the pet. ether extract of *S. aquifolium* and the aqueous extract of *G. rugosa*, which showed moderate antiviral effects. Both pigment extracts of *S. aquifolium* and *G. rugosa* demonstrated intermediate levels of antiviral activity. In contrast, the aqueous extract of *S. aquifolium* displayed the lowest antiviral activity against SARS-CoV-2.

#### In-vitro anticancer activity

Data summarized in Supplementary Tables 11–13 indicate that the aqueous and pigment extracts of *S. aquifolium* exhibited promising cytotoxic activity against A549 lung cancer cells, with inhibition percentages of 70.00% and 62.56%, respectively. Similarly, the pet. ether, aqueous, and pigment extracts of *G. rugosa* demonstrated moderate anticancer activity against the same cell line, achieving inhibition rates of 44.03%, 55.12%, and 48.11%, respectively, when compared to the standard drug doxorubicin. Furthermore, both aqueous extracts of *S. aquifolium* and *G. rugosa* displayed significant cytotoxic effects against HCT116 colon cancer cells, with inhibition percentages of 76.02% and 61.17%, respectively. These findings highlight the potential of aqueous extracts from both macroalgae as promising candidates for anticancer therapy. On the other hand, weak activity was observed against MCF7 breast cancer cells for all tested extracts. Importantly, all macroalgal extracts exhibited concentration-dependent cytotoxic effects across the tested cancer cell lines.

### Computer-guided docking study of the isolated compounds

#### Molecular dynamic and system stability

A molecular dynamics simulation was conducted to forecast the behavior of isolated compounds when they bind to the active site of a protein, as well as to evaluate their interaction and stability through simulation^71^. This investigation utilized Root-Mean-Square Deviation (RMSD) to assess the stability of the systems during the 40 ns simulations. The average RMSD values recorded for all frames of the systems were 1.577 ± 0.28 Å for the Apo and 1.35 ± 0.21 Å for the Phytol – Mpro complex, as shown in Supplementary Fig. 6A. For the Apo and Phytol – VEGFR2 complex, the RMSD values were 1.36 ± 0.17 Å and 1.12 ± 0.11 Å, respectively, as illustrated in Supplementary Fig. 7a. These findings indicated that the Phytol-bound protein complex system achieved a comparatively more stable conformation than the other systems examined.

In the course of MD simulation, it is essential to evaluate the structural flexibility of proteins in response to ligand binding, as this is crucial for understanding the behavior of residues and their interactions with the ligand^72^. The fluctuations of protein residues were analyzed using the Root-Mean-Square Fluctuation (RMSF) algorithm to assess the impact of inhibitor binding on the respective targets over a duration of 200 ns simulations. The average RMSF values obtained were 1.01 ± 0.39 Å and 0.99 ± 0.38 Å for the Apo and Phytol – Mpro complex, as illustrated in Supplementary Fig. 6B, while for the Apo and Phytol – VEGFR2 complex, the values were 0.94 ± 0.48 Å and 0.86 ± 0.45 Å, respectively, as shown in Supplementary Fig. 7b. These results indicate that the Phytol-bound protein complex system exhibits greater residue fluctuation compared to the other systems.

ROG aimed to assess the overall compactness of the system as well as its stability upon ligand binding during molecular dynamics (MD) simulation^73,74^. The average radius of gyration (Rg) values were recorded as 22.30 ± 0.11 Å and 22.33 ± 0.09 Å for the Apo and Phytol – Mpro complex, respectively, as shown in Supplementary Fig. 6C. For the Apo and Phytol – VEGFR2 complex, the average Rg values were 19.93 ± 0.09 Å and 19.90 ± 0.07 Å, respectively, as illustrated in Supplementary Fig. 7c. Based on the observed behavior, the Phytol compound exhibits a highly flexible structure in relation to the Mpro and VEGFR2 receptors.

The density of the protein’s hydrophobic core was analyzed through the calculation of the solvent accessible surface area (SASA) of the protein. This analysis involved measuring the surface area of the protein that is exposed to the solvent, a factor that is crucial for the stability of biomolecules^75^. The average SASA values were 14021.99Å, and 13821.14 Å, for Apo, and Phytol – M^pro^ complex, Supplementary Fig. 6D, 14922.24Å, and 14550.78 Å, for Apo, and Phytol – VEGFR2 complex, respectively Supplementary Fig. 7d. The SASA finding, in conjunction with the observations derived from the RMSD, RMSF, and ROG computations, validated that the Salvimulticanol complex system remains intact within the catalytic domain binding site of the Mpro and VEGFR2 receptor.

#### Binding interaction mechanism based on binding free energy calculation

A popular method for determining the free binding energies of small molecules to biological macromolecules is the molecular mechanics energy technique (MM/GBSA), which combines the generalized Born and surface area continuum solvation, and it may be more trustworthy than docking scores^76^. The MM-GBSA program within AMBER18 was employed to compute the binding free energies by extracting snapshots from the system trajectories. As indicated in Supplementary Table 14, all calculated energy components reported (with the exception of ΔGsolv) exhibited significantly negative values, suggesting favorable interactions.

A comprehensive examination of each individual energy contribution that leads to the reported binding free energies reveals that the interactions between the Phytol compound and the Mpro and VEGFR2 receptor protein residues are primarily influenced by the more positive Van der Waals energy components, as demonstrated in Supplementary Tables 15 and 16, respectively.

#### Identification of the critical residues responsible for ligands binding

To gain further insight into the key residues that play a role in the inhibition of the Mpro receptor by phytol compounds, the total energy associated with the interaction of phytol compounds with these enzymes was analyzed in terms of specific site residues. Figure 8A indicates that the primary favorable contribution of phytol compounds to the Mpro receptor is largely attributed to residues Gln 19 (−0.102 kcal/mol) and Thr 25 (−0.698 kcal/mol), Leu 27 (−1.018 kcal/mol), Asn 28 (−0.197 kcal/mol), Hie 41 (−1.222 kcal/mol), Val 42 (−0.176 kcal/mol), Tyr 118 (−0.717 kcal/mol), Asn 119 (−0.362 kcal/mol), Asn 142 (−0.286 kcal/mol), Gly 143 (−1.321 kcal/mol), Ser 144 (−0.262 kcal/mol), Cys 145 (−0.897 kcal/mol), Met165 (−0.959 kcal/mol), Asp 187 (−0.67 kcal/mol), Arg188 (−0.34 kcal/mol), and Gln 189 (−1.224 kcal/mol).

On the other hand, the major favorable contribution of phytol compound to the ATP binding site receptor of VEGFR2 receptor is predominantly observed from residues Pro 6 (−1.78 kcal/mol), Leu 7 (−0.9 kcal/mol), Leu 34 (−0.439 kcal/mol), Val42 (−0.759 kcal/mol), Ala 60 (−0.513 kcal/mol), Val 61 (−0.177 kcal/mol), Lys 62 (−1.071 kcal/mol), Leu 82 (−1.775 kcal/mol), Leu 83 (−1.379 kcal/mol), Ile 86 (−0.756 kcal/mol), Val 92 (−0.675 kcal/mol), Val 93 (−1.258 kcal/mol), Val 108 (−0.449 kcal/mol), Val 110 (−1.173 kcal/mol), Cys 161 (−1.562 kcal/mol), Ile 162 (−2.021 kcal/mol), Hie 163 (−1.208 kcal/mol), Arg 164 (−0.337 kcal/mol), Leu 172 (−0.601 kcal/mol), Ile 181 (−0.71 kcal/mol), Cys 182 (−1.084 kcal/mol), Asp 183 (−0.885 kcal/mol) and Phe 184 (−0.934 kcal/mol). Supplementary Fig. 8B.

### Ligand–residue interaction network profiles

#### Docked Mpro complexes

The docked phytol- M^pro^ complex showed that the phytol has established a secure H-bonding interaction with Cys 44. In addition, the phytol has formed api-alkyl interaction with residues Hie 44, Met 49, and Cys145 as shown in Supplementary Fig. 9.

#### Docked VEGFR2 complex

The docked VEGFR2–phytol complex showed that the phytol has formed api-alkyl interaction with Lys 62, Leu 83, Val 93, Val 110, and Cys 182. It is worthy to note that api-alkyl and Van der Waal interactions have been created between the phytol and the pharmacoporic hot spot residue Hie 163.

## Discussion

The pet. ether extract of *S. aquifolium* was found to be rich in sterols, particularly desmosterol and fucosterol, consistent with the findings of Dhargalkar and Pereira^12^. It also contained notable amounts of fatty alcohols, especially phytol, and fatty acids such as hexadecanoic acid, corroborating the observations of Rushdi et al.^10^. Additionally, it exhibited significant levels of polysaccharides primarily composed of rhamnose and galactose, essential amino acids such as threonine and histidine, and non-essential ones like aspartic acid. Pigment analysis confirmed the abundance of fucoxanthin and sterols, in line with prior reports by Dhargalkar and Pereira^12^ and Sohn et al.^77^. While protein content was low, the species was notably rich in carbohydrates and β-carotene, as also noted by El-Beltagi et al.^78^.

On the other hand, *G. rugosa* was found to contain a considerable amount of fatty acids and proteins, including essential amino acids like histidine and non-essential ones such as aspartic acid and tyrosine. Its polysaccharide profile was predominantly composed of mannose and arabinose. Notably, it exhibited high pigment content, especially erythrin, aligning with findings reported by Dodds and Whiles^13^.

Significant antioxidant and antiviral activities were observed in the pet. ether extracts of both species, as well as in the aqueous extract of *G. rugosa*. Moreover, notable anticancer activity was demonstrated by the aqueous extracts of both algae and the pigment extract of *S. aquifolium*. The observed biological activities of the pet. ether extracts can be attributed to their high content of fatty acids (FAs) and phytosterols. FAs act as energy storage molecules and signaling compounds, modulating gene expression, cellular differentiation, and development^79^. Their biological functions depend on the degree of unsaturation. Fucosterol, in particular, was reported to exert antioxidant effects by upregulating antioxidant enzymes such as glutathione peroxidase, superoxide dismutase, and catalase^80^.

Regarding the antioxidant, antiviral, and anticancer effects of the aqueous extracts, these can largely be attributed to the presence of polyuronic polysaccharides, which are structurally distinct from those found in terrestrial plants. These polysaccharides are widely recognized for their biological activities—including antioxidant, anticancer, anti-inflammatory, anti-diabetic, anticoagulant, immunomodulatory, and anti-HIV effects—and have diverse applications in pharmaceutical, nutraceutical, and cosmetic industries. Notably, low molecular weight polysaccharides exhibit stronger antioxidant potential due to their higher proton-donating capacity. In red algae, cell walls contain biologically active polysaccharides such as galactose, glucose, rhamnose, glucuronic acid, and arabinose, which possess antiviral and anticancer properties. These polysaccharides can inhibit viral entry by preventing the formation of virus-cell surface complexes^78^. Furthermore, polysaccharides from *Sargassum* spp. have demonstrated antiviral effects against HSV-1 and HSV-2^19^.

Proteins extracted from the aqueous fractions were found to contain all essential and non-essential amino acids. Seaweed-derived proteins and peptides are known to possess strong antioxidant activities, particularly those of low molecular weight^78^. The presence of cystine likely contributes to this antioxidant activity, as sulfur-containing and aromatic amino acids (e.g., Trp, Tyr, Phe, Cys, Met) are particularly effective radical scavengers. In this study, glutamic acid, cysteine, and glycine—precursors of glutathione (GSH), hydrogen sulfide, and taurine—were detected and are associated with strong antioxidant and neuroprotective activities. Cysteine’s sulfhydryl group undergoes oxidation under oxidative stress, forming reversible or irreversible oxidation products such as disulfides, sulfenic, sulfinic, or sulfonic acids. GSH plays a dual role as both a direct antioxidant and a modulator of neuronal redox homeostasis^28^.

Carotenoids, another vital group of antioxidants found in both species, protect against oxidative stress by quenching singlet oxygen and other reactive species. The presence of allenic bonds and additional oxygen-containing functional groups in fucoxanthin and astaxanthin enhances their free radical scavenging ability^69^. Lipid-soluble carotenoids such as lutein, lycopene, and β-carotene are efficient quenchers of singlet oxygen, which is known to cause DNA damage. These pigments can absorb the energy of singlet oxygen, thereby protecting cellular structures. Carotenoids like lutein and diatoxanthin, identified in our study, owe their antioxidant potential to their conjugated double bonds and functional groups^70^. Fucoxanthin has shown multiple biological effects, including inhibition of DNA fragmentation, antiproliferative activity against various cancer cell lines (e.g., HCT-116, MCF-7, HepG2), and modulation of enzymes like PARP and caspases. It also demonstrated antiviral activity against HIV-1, HSV-1, HSV-2, and ECHO-1. β-carotene also showed antiproliferative and antioxidant properties in murine osteosarcoma models^78^.

Terpenoids also demonstrated promising anticancer properties. These compounds have been widely reported as potential chemopreventive and chemotherapeutic agents^81^. In particular, sargachromanols in the pigment extract of *S. aquifolium* showed strong antioxidant activity in the DPPH assay^1^. Phytol, a diterpenoid found in both species, possesses diverse pharmacological activities, including anticancer, antioxidant, and immunomodulatory properties. It has been reported to induce DNA repair and apoptosis in breast cancer cells, as well as to scavenge free radicals through its alcohol group, which contributes to the formation of a stabilized radical resonance structure^82,83^.

In our study, the antiviral and anticancer properties of *S. aquifolium* and *G. rugosa* were regarded mainly to the presence of phytol. Docking analysis was used to guarantee this conclusion. The residues Gln 19, Thr25, Leu 27, Asn 28, Hie 41, Val 42, Tyr 118, Asn 119, Asn 142, Gly 143, Ser 144, Cys 145, Met165, Asp 187, Arg188, and Gln 189 are primarily responsible for the phytol compound’s major beneficial contribution to the M pro receptor (which is specific for the SARS-COV2 virus). Phytol has been shown to have a secured H-bonding contact with Cys 44 and to create api-alkyl interactions with residues Hie 44, Met 49, and Cys 145. This outcome is demonstrated by the docked Phytol-Mpro complex.

However, residues Pro 6, Leu 7, Leu 34, Val 42, Ala 60, Val 61, Lys 62, Leu 82, Leu 83, Ile 86, Val 92, Val 93, Val 108, Val 110, Cys 161, Ile 162, Hie 163, Arg 164, Leu 172, Ile 181, Cys 182, Asp 183, and Phe 184 are primarily responsible for the phytol compound’s significant positive contribution to the VEGFR2 receptor’s ATP binding site receptor, which is typical of cancer cells. The phytol has established api-alkyl interactions with Lys 62, Leu 83, Val 93, Val 110, and Cys 182, according to the docked VEGFR2-Phytol complex. Notably, the phytol and the pharmacoporic hot spot residue Hie 163 have developed Vander Waal and api-alkyl interactions.

## Conclusion

The pet. ether extracts of *Sargassum aquifolium* and *Galaxaura rugosa*, as well as the aqueous extract of *G. rugosa*, exhibited notable in vitro antioxidant and antiviral activities against SARS-CoV-2. The pet. ether extract of *G. rugosa* showed strong antiviral potency (IC₅₀ = 6.454 µg/mL), comparable to remdesivir. Furthermore, the aqueous extracts of both macroalgae, along with the pigment extract of *S. aquifolium*, showed significant in vitro cytotoxic activity against lung and colon cancer cell lines. Notably, the aqueous extract of *S. aquifolium* inhibited 76.02% of colon cancer cells, its pigment extract inhibited 62.56% of lung cancer cells, and the aqueous extract of *G. rugosa* inhibited 61.17% of colon cancer cells. These biological results are supported by phytochemical profiling, which revealed sterols, fatty acids, terpenoids, carotenoids, sugars, and amino acids, with phytol highlighted in docking studies as a likely contributor. This study provides the first comprehensive phytochemical and biological characterization of these two macroalgal species, supporting their potential as natural sources of antioxidant, antiviral, and anticancer agents, and warranting further in vivo and clinical investigations.

## Supplementary Information

Below is the link to the electronic supplementary material.

Supplementary Material 1

## Data Availability

The datasets used and/or analysed during the current study available from the corresponding author on reasonable request.

## Abbreviations

*S. aquifolium*: *Sargassum aquifolium*
G. rugosa: Galaxaura rugosa
Asp.: Aspartic acid
Glu.: Glutamic acid
Ser.: Serine
Gly.: Glycine
His.: Histidine
Arg.: Arginine
Thr.: Threonin
Ala.: Alanine
Pro.: Proline
Tyr.: Tyrosine
Val.: Valine
Met.: Methionine
Cys.: Cysteine
Ile.: Isoleucine
ABTS: 2,2’-azinobis-3-ethylbenzo thizoline-6-sulfonate
DMSO: 7- Dimethyl sulfoxide
Pet. Ether: Petroleum ether
Leu.: Leucine
Phe: Phenylalanine
Lys: Lysine
Trp: Tryptophan
SPS: Sulphated polysaccharides
SHP-1: Protein tyrosine phosphatase non‐receptor type 6
MDA: Malondialdehyde
APOP: Protein oxidation product
NO: Nitric oxide
GSH: Gutathione
CAT: Catalase
GST: Glutathione S-transeferase
Bcl-xL: Antiapoptotic factor
PARP: Poly-ADP‐ribose polymerase
VCAM-1, ICAM‐1: Genes coding for vascular adhesion proteins
DPPH: 1,1-diphenyl-2-picryl hydrazyl
DMEM: Dulbecco΄s Modified Eagle Medium, Gibco- BRL
MTT: 3-(4,5-dimethylthiazol-2-yl)-2,5-diphenyltetrazolium bromide
