# Supplementary material for: Targeting cancer and SARS-CoV-2: phytochemical, biological and molecular dynamic investigations of Sargassum aquifolium and Galaxaura rugosa
Source: Sci Rep. 2025 Nov 11;15:39417. doi: 10.1038/s41598-025-22987-z (PMC12606105; doi:10.1038/s41598-025-22987-z)
Supplement: Supplementary file 1 — Supplementary Material 1 [file 41598_2025_22987_MOESM1_ESM.docx]

**Targeting Cancer and SARS-CoV-2: Phytochemical, Biological and Molecular Dynamic Investigations of *Sargassum aquifolium* and *Galaxaura rugosa***

**Asmaa S. Abd Elsamiae^1^, Abdel Nasser B. Singab^2^, Ataa Said^1^, Omayma A. Eldahshan^2^, Nada M. Mostafa^2^, Mohamed S. Elnaggar^2^,** [**Asmaa F. Aboul Naser**](https://www.tandfonline.com/author/Aboul+Naser%2C+Asmaa+F)**^3^, Abo El-Khair B El-Sayed^4^, Ahmed A. El‑Rashedy^5^, Omnia M Kutkat^6^,** [**Amal M. El-Feky**](https://www.tandfonline.com/author/El-Feky%2C+Amal+M)**^1^ ***

^1^ Pharmacognosy Department, National Research Center, 33 El Bohouth St. (Former El Tahrir St.), Dokki, P.O. 12622, Giza, Egypt

^2^ Department of pharmacognosy, Faculty of pharmacy, Ain Shams University, Cairo 11566, Egypt.

^3^ Department of Therapeutic Chemistry, National Research Center, 33 El Bohouth St. (Former El Tahrir St.), Dokki, P.O. 12622, Giza, Egypt

^4^ Algal Biotechnology Unit, Biological and Agricultural Research Division, National Research Centre, Dokki, Giza, Egypt

^5^ Natural and Microbial Products Department, National Research Center, 33 El Bohouth St. (Former El Tahrir St.), Dokki, P.O. 12622, Giza, Egypt

6 Department of Water Pollution Research, Center of Scientific Excellence for Influenza Virus, National Research Centre, Dokki, Giza, Egypt

**For Correspondence: ammelfeky@hotmail.com*

Supplementary Table 1: GC/MS results of the USM of *S. aquifolium* and *G. rugosa.*

| **Peak no.** | **Identified compound** | **Rt** | **Ret. Index**  **lit.** | **Ret. Index**  **exp.** | **Chemical formula** | **MWt** | **Base**  **peak** | **Area%** | |
| --- | --- | --- | --- | --- | --- | --- | --- | --- | --- |
|  |  |  |  |  |  |  |  | ***S. aquifolium*** | ***G. rugosa*** |
|  | 1-Methyl-1-propylpentyl benzene | 24.19 | 1504 | 1507 | C_15_H_24_ | 204 | 105 | 0.43 | - |
|  | 1-Butylhexyl benzene | 24.59 | 1624 | 1622 | C_16_H_26_ | 218 | 91 | 0.24 | 0.47 |
|  | 1-Propylheptyl benzene | 24.80 | 1624 | 1622 | C_16_H_26_ | 218 | 91 | 0.21 | 0.39 |
|  | 1-Ethyloctyl benzene | 25.25 | 1624 | 1622 | C_16_H_26_ | 218 | 91 | 0.25 | 0.52 |
|  | 1-Methylnonyl benzene | 26.14 | 1624 | 1622 | C_16_H_26_ | 218 | 105 | 0.41 | 0.81 |
|  | 1-Pentylhexyl benzene | 26.89 | 1724 | 1721 | C_17_H_28_ | 232 | 91 | 0.49 | 0.79 |
|  | 1-Butylheptyl benzene | 26.98 | 1724 | 1721 | C_17_H_28_ | 232 | 91 | 0.80 | 1.65 |
|  | 1-Propyloctyl benzene | 27.21 | 1724 | 1721 | C_17_H_28_ | 232 | 91 | 1.67 | 1.99 |
|  | 1-Ethylnonyl benzene | 27.70 | 1724 | 1721 | C_17_H_28_ | 232 | 91 | 3.24 | 3.48 |
|  | 1-Methyldecyl benzene | 28.57 | 1724 | 1721 | C_17_H_28_ | 232 | 105 | 6.89 | 4.58 |
|  | 1-Pentylheptyl benzene | 29.16 | 1823 | 1825 | C_18_H_30_ | 246 | 91 | 5.93 | 4.08 |
|  | 1-Butyloctyl benzene | 29.25 | 1823 | 1825 | C_18_H_30_ | 246 | 91 | 5.91 | 3.91 |
|  | 1-Propylnonyl-benzene | 29.52 | 1823 | 1825 | C_18_H_30_ | 246 | 91 | - | 5.11 |
|  | 1-Methylundecyl benzene | 29.83 | 1823 | 1825 | C_18_H_30_ | 246 | 91 | 13.78 | 11.17 |
|  | 1-Ethyldecyl benzene | 30.02 | 1823 | 1825 | C_18_H_30_ | 246 | 91 | 8.39 | 7.32 |
|  | 1-Nonadecene | 30.99 | 1900 | 1904 | C_19_H_32_ | 266 | 97 | 0.56 | - |
|  | 1-Pentyloctyl benzene | 31.33 | 1922 | 1926 | C_19_H_32_ | 260 | 91 | 9.12 | 8.84 |
|  | 1-Butylnonyl benzene | 31.47 | 1922 | 1926 | C_19_H_32_ | 260 | 91 | 6.55 | 6.32 |
|  | 1-Methyldodecyl benzene | 31.58 | 1922 | 1926 | C_19_H_32_ | 260 | 105 | 11.34 | 15.25 |
|  | 1-Propyldecyl benzene | 31.74 | 1922 | 1926 | C_19_H_32_ | 260 | 91 | 6.44 | 7.12 |
|  | 1-Ethylundecyl benzene | 32.24 | 1922 | 1926 | C_19_H_32_ | 260 | 91 | 7.96 | 10.59 |
|  | 1-Eicosene | 33.50 | 1999 | 2003 | C_20_H_40_ | 280 | 43 | - | 0.30 |
|  | Phytol, acetate | 34.02 | 2168 | 2175 | C_22_H_42_O_2_ | 338 | 71 | 0.17 | - |
|  | 1-Tetracosene | 34.68 | 2397 | 2403 | C_24_H_48_ | 336 | 57 | - | 0.56 |
|  | 3.α., 11. β.-dihydroxy-, 3-acetate-(alpha.-Androstan-17-one) | 38.23 | 2456 | 2464 | C_21_H_32_O_4_ | 348 | 69 | 0.17 | - |
|  | 1-Hexacosene | 38.47 | 2596 | 2590 | C_26_H_52_ | 364 | 97 | 0.24 | 0.28 |
|  | Cholesterol | 52.99 | 2596 | 2603 | C_27_H_46_O | 386 | 43 | 0.39 | 0.07 |
|  | Fucosterol | 56.01 | 2780 | 2786 | C_29_H_48_O | 412 | 314 | 1.60 | - |
|  | 1-Heptacosanol | 56.97 | 2948 | 2957 | C_27_H_56_O | 396 | 97 | 0.33 | - |
|  | Methyl, 2, 3-dibenzoate 4-*p*-toluenesulfonate, β-L-arabinopyranoside | 57.02 | 3979 | 3987 | C_27_H_26_O_9_S | 526 | 105 | 0.10 | - |

**Ret. Index lit.=Retention index literature, Ret. Index exp.=Retention index experimental.**

Supplementary Table 2: GC/MS results of the FAME of *S. aquifolium* and *G. rugosa.*

| **Peak no.** | **Identified compound** | **Rt** | **Ret. Index**  **lit.** | **Ret. Index**  **exp.** | **Chemical formula** | **MWt** | **Base**  **peak** | **Area%** | |
| --- | --- | --- | --- | --- | --- | --- | --- | --- | --- |
|  |  |  |  |  |  |  |  | ***S. aquifolium*** | ***G. rugosa*** |
|  | Methyl tetradecanoate | 29.01 | 1680 | 1678 | C_15_H_30_O_2_ | 242 | 74 | 3.06 | - |
|  | Hexadecanoic acid, methyl ester | 33.36 | 1878 | 1879 | C_17_H_34_O_2_ | 270 | 74 | 25.67 | 0.57 |
|  | n-Hexadecanoic acid | 34.17 | 1878 | 1881 | C_17_H_34_O_2_ | 270 | 43 | - | 0.16 |
|  | (Z)-7-Hexadecenoic acid, methyl ester | 34.56 | 1886 | 1888 | C_17_H_32_O_2_ | 268 | 55 | - | 0.19 |
|  | Hexadecanoic acid, trimethylsilyl ester | 35.75 | 1987 | 1989 | C_19_H_40_O_2_Si | 328 | 117 | - | 0.11 |
|  | 10-Heptadecen-8-ynoic acid, methyl ester | 35.88 | 2003 | 2000 | C_18_H_30_O_2_ | 278 | 79 | - | 0.15 |
|  | Methyl stearate | 36.27 | 2077 | 2075 | C_19_H_38_O_2_ | 298 | 74 | 2.17 | 0.57 |
|  | 11-Octadecenoic acid, methyl ester | 36.68 | 2085 | 2083 | C_19_H_36_O_2_ | 296 | 55 | 5.69 | - |
|  | 10-Octadecenoic acid, methyl ester | 36.79 | 2085 | 2087 | C_19_H_36_O_2_ | 296 | 41 | 17.37 | 0.37 |
|  | (Z)-9-Octadecenoic acid, methyl ester | 36.81 | 2085 | 2089 | C_19_H_36_O_2_ | 296 | 55 | - | 0.60 |
|  | 9,12-Octadecadienoic acid, methyl ester | 37.06 | 2093 | 2098 | C_19_H_34_O_2_ | 294 | 67 | 2.89 | 0.20 |
|  | Oxiraneoctanoic acid, 3-octyl-, methyl ester | 38.73 | 2129 | 2123 | C_19_H_36_O_3_ | 312 | 69 | - | 0.10 |
|  | cis-10-Nonadecenoic acid, methyl ester | 38.98 | 2185 | 2190 | C_20_H_38_O_2_ | 310 | 55 | - | 0.05 |
|  | Methyl 18-methylnonadecanoate | 39.90 | 2212 | 2206 | C_21_H_42_O_2_ | 326 | 74 | - | 0.15 |
|  | Tetramethylheptadecan-4-olide | 40.41 | 2258 | 2252 | C_21_H_40_O_2_ | 324 | 99 | - | 0.10 |
|  | cis-Methyl 11-eicosenoate | 40.46 | 2284 | 2278 | C_21_H_40_O_2_ | 324 | 55 | - | 0.99 |
|  | cis-13-Eicosenoic acid, methyl ester | 41.57 | 2284 | 2290 | C_21_H_40_O_2_ | 324 | 55 | - | 0.13 |
|  | Docosanoic acid, methyl ester | 43.95 | 2475 | 2469 | C_23_H_46_O_2_ | 354 | 74 | - | 0.14 |
|  | 13-Docosenoic acid, methyl ester | 44.03 | 2483 | 2489 | C_23_H_44_O_2_ | 352 | 55 | - | 0.06 |
|  | Hexadecanoic acid, 2-hydroxy-1-(hydroxymethyl)ethyl ester | 44.89 | 2498 | 2502 | C_19_H_38_O_4_ | 330 | 43 | - | 0.13 |
|  | Octan-2-yl palmitate | 45.80 | 2510 | 2516 | C_24_H_48_O_2_ | 368 | 57 | - | 0.46 |
|  | 1,2-Benzenedicarboxylic acid, dinonyl ester | 47.995 | 3031 | 3037 | C_26_H_42_O_4_ | 418 | 149 | - | 29.14 |
|  | 1,2-Benzenedicarboxylic acid, bis(8-methylnonyl) ester | 49.873 | 3101 | 3108 | C_28_H_46_O_4_ | 446 | 149 | - | 2.67 |

Ret. Index lit.=Retention index literature, Ret. Index exp.=Retention index experimental.

Supplementary Table 3: Monosugar content of the macroalgal isolated polysaccharides.

| **Macroalgae** | Relative % | | | | | | | | |
| --- | --- | --- | --- | --- | --- | --- | --- | --- | --- |
|  | Rhamnose | Galactose | Glucose | Arabinose | Mannitol | Ribose | Xylose | Mannose | Sorbitol |
| *S. aquifolium* | 21.75 | 13.68 | 7.96 | 2.69 | 1.71 | 1.25 | 0.89 | - | - |
| *G. rugosa* | 2.02 | - | 11.44 | 22.55 | 0.76 | 0.31 | 5.83 | 37.40 | 1.55 |

Supplementary Table 4: Amino acid analysis of the prepared proteins of both macroalgae *S. aquifolium* and *G. rugosa*.

| **Essential amino acids** | | | | | | | | | |
| --- | --- | --- | --- | --- | --- | --- | --- | --- | --- |
|  | His. | Thr. | Val. | Met. | Ile. | Leu. | Phe. | Lys. | Tryp. |
| ***S. aquifolium*** | 93.40 | 136.03 | 26.09 | 52.23 | 11.46 | 14.45 | 49.99 | 59.22 | not determined |
| ***G. rugosa*** | 122.38 | 58.95 | 41.04 | 76.79 | 31.00 | 18.47 | 27.24 | 21.89 |  |
| **Non-essential amino acids** | | | | | | | | | |
|  | Asp. | Glu. | Ser. | Gly. | Arg. | Cys. | Ala. | Pro. | Tyr. |
| ***S. aquifolium*** | 96.48 | 86.28 | 80.41 | 34.03 | 56.68 | 5.77 | 38.69 | 35.26 | 71.78 |
| ***G. rugosa*** | 88.21 | 73.39 | 85.87 | 36.24 | 47.07 | 8.97 | 54.32 | 60.86 | 97.87 |

Supplementary Table 5: Quantitative estimation of natural pigments in *S. aquifolium* and *G. rugosa*.

| **Pigment** | **Conc. (µg/ml)** | | | | |
| --- | --- | --- | --- | --- | --- |
|  | **Chlorophyll a** | **Chlorophyll b** | **Total chlorophyll** | **Carotenoids** | **Pigment index** |
| *S. aquifolium* | 11.49 | 25.96 | 33.46 | 7.54 | 0.66 |
| *G. rugosa* | 12.53 | 17.33 | 26.11 | 2.87 | 0.23 |

Supplementary Table 6: LC/MS-MS of the pigment extract of *S. aquifolium* using positive and negative ion acquisition modes.

| **No.** | **Tentative identification** | **M.wt** | **[M+H]^+^**  **(Rt)** | **[M-H]^-^**  **(Rt)** | **Characteristic fragments** | **Chemical formula** | **Class** | **Ref.** |
| --- | --- | --- | --- | --- | --- | --- | --- | --- |
|  | Loliolide | 196.2 | - | 195.2  (0.80) | 179, 133, 105, 91 | [C_11_H_16_O_3_](https://pubchem.ncbi.nlm.nih.gov/#query=C11H16O3) | Terpenes | **Saraswati *et al*., 2019** |
|  | β-sitosterol | 414.7 | 415.7  (13.03) | 413.7  (29.82) | 396, 329, 303, 273 | C_29_H_50_O | Sterol | **Hannan et al., 2020** |
|  | Fucoxanthin | 658.6 | 659.6  (15.15) | 657.6  (25.95) | 640, 148,108, 82 | C_42_H_28_O_6_ | Pigment | **Pereira *et al*., 2021** |
|  | β -carotene | 536.4 | - | 535.4  ( 15.71) | 444, 135, 429, 201,241,267,293 | C_40_H_56_ | Pigment | **Ibrahim *et al*., 2021a;Cikoš et al., 2022; Haugan, 1994** |
|  | (5E,9E,13E)-6,10,14-trimethyl pentadeca-5,9,13-trien-2,12-dione | 275 | 276  (15.80) | - | 219 | C_18_H_28_0_2_ | Terpenes | **Ryu et al., 2003** |
|  | Ergosterol | 396.6 | 397.6  (15.84) | - | 312, 294, 270, 191, 153 | C_28_H_44_O | Sterol | **Hannan et al., 2020** |
|  | Brassicasterol | 398.7 | 399.7  ( 15.94) | 397.7  (16.44) | 380, 99 | C_28_H_46_O | Sterol | **Sohn et al., 2021; Hannan et al., 2020** |
|  | β- zeacarotene | 538 | 539  (16.43) | - | 400, 308,176,118 | C_40_H_58_ | Pigment | **Cikoš et al., 2022** |
|  | Desmosterol | 384.6 | 385.6  (16.53) | 383.6  (22.91) | 368,365, 350, 298, 270, 254, 252, 228, 212 | C_27_H_44_O | Sterol | **Lopes et al., 2013** |
|  | Tuberatolide B | 422.6 | 423.6  (16.66) | 421.6  (27.01) | 404, 358 | [C_27_H_34_O_4_](https://pubchem.ncbi.nlm.nih.gov/#query=C27H34O4) | Terpenes | **Saraswati *et al*., 2019; Kwon *et al*.,2022** |
|  | Erythrin | 422.4 | 423.4  (16.76) | 421.4  (27.01) | 404, 386, 340 | C_20_H_22_O_10_ | Pigment | **Dodds and Whiles, 2020** |
|  | Saringosterone | 426 | 427  (17.11) | - | 269, 382 [M-C3H7], 313, 271 | C_29_H_46_O_2_ | Sterol | **Ayyad et al., 2011** |
|  | Sargahydroquinoic acid | 426.7 | 427.7  (17.26) | - | 408, 390, 344 | [C_27_H_38_O_4_](https://pubchem.ncbi.nlm.nih.gov/#query=C27H38O4) | Terpenes | **Saraswati *et al*., 2019; Kwon *et al*.,2022** |
|  | Campestanol | 402.7 | 403.7  (18.23) | 401.7  (19.08) | 233, 219 | [C_28_H_50_O](https://pubchem.ncbi.nlm.nih.gov/#query=C28H50O) | Sterol | **Kircher and Rosenstein, 1974** |
|  | Cyclosadol | 440.7 | 441.7  (18.31) | - | 422 | [C_31_H_52_O](https://pubchem.ncbi.nlm.nih.gov/#query=C31H52O) | Terpenes | **Dembitsky *et al*., 2021** |
|  | Pelargonidin | 271.2 | 272.2  (18.39) | 270.2  (25.43) | 253, 235 | C_15_H_11_O_5_ | Pigment | **Haugan, 1994** |
|  | Sargaquinoic acid | 424.6 | 425.6  (18.42) | - | 378 | C_27_H_36_O_4_ | Terpenes | **Saraswati *et al*., 2019 ,18; Kwon *et al*.,2022** |
|  | 23(E)-25-methoxy cycloart-23-en-3β-ol | 455 | 456  (19.10) | - | 437 | C_31_H_52_O_2_ | Terpenes | **Ryu et al., 2003** |
|  | (5E,10Z)-6,10,14-trimethyl pentadeca-5,10-dien-2,12-dione | 277 | 278  (19.31) | 277.4  (19.36) | 221 | C_18_H_30_O_2_ | Terpenes | **Ryu et al., 2003** |
|  | cryptoxanthin | 552 | 553  (19.34) | 551  (16.44) | 534, 496, 460 | C_40_H_56_O | Pigment | **Haugan, 1994** |
|  | Labdane | 278.5 | 279.5  (19.36) | 277.5  (19.36) | 180, 98 | [C_20_H_38_](https://pubchem.ncbi.nlm.nih.gov/#query=C20H38) | Terpenes | **Saraswati *et al*., 2019** |
|  | Phytol | 296.5 | 297.5  (19.92) | 295.5  (15.42) | 281, 265, 253 | [C_20_H_40_O](https://pubchem.ncbi.nlm.nih.gov/#query=C20H40O) | Terpenes | **Rushdi et al., 2020** |
|  | Prostane | 280.5 | 281.5  (20.44) | 279.5  (20.83) | 170, 76 | [C_20_H_40_](https://pubchem.ncbi.nlm.nih.gov/#query=C20H40) | Terpenes | **Kwon *et al*.,2022** |
|  | Adonirubin | 580.5 | 581.5  (20.48) | - | 562, 91 | C_40_H_52_O_3_ | Pigment | **Pereira *et al*., 2021** |
|  | Sargachromanol E | 428.6 | 429.6  (20.84) | - | 410, 392 | [C_27_H_40_O_4_](https://pubchem.ncbi.nlm.nih.gov/#query=C27H40O4) | Terpenes | **Saraswati *et al*., 2019** |
|  | Saringosterol | 428.7 | 429.7  (20.87) | - | 410, 382 | [C_29_H_48_O_2_](https://pubchem.ncbi.nlm.nih.gov/#query=C29H48O2) | Sterol | **Ikekawa *et al*., 1968; Hannan et al., 2020; Ayyad et al., 2011** |
|  | Uvaol | 442.7 |  | 441.7  ( 20.92) | 424, 192, 163, 109, 95 | [C_30_H_50_O_2_](https://pubchem.ncbi.nlm.nih.gov/#query=C30H50O2) | Terpenes | **Kwon *et al*.,2022** |
|  | Cycloartenol | 426.7 | 427.7  (22.58) | - | 408, 69 | [C_30_H_50_O](https://pubchem.ncbi.nlm.nih.gov/#query=C30H50O) | Sterol | **Dembitsky *et al*., 2021** |
|  | Asiatic acid | 488.7 | 489.7  (22.61) | - | 410, 422, 380 | [C_30_H_48_O_5_](https://pubchem.ncbi.nlm.nih.gov/#query=C30H48O5) | Terpenes | **Kwon *et al*.,2022** |
|  | Canthaxanthin | 564 | 565  (22.67) | - | 546, 458, 362, 202, 190, 132 | C_40_H_52_O_2_ | Pigment | **Pereira *et al*., 2021** |
|  | Diatoxanthin | 566 | 567  (22.77) | - | 549,443,217,199,310,175,145 | C_40_H_54_O_2_ | Pigment | **Ibrahim *et al*., 2021a; Balasubramaniam et al., 2020, Haugan, 1994** |
|  | Campesterol | 400.7 | 401.7  (24.49) | 399.7  (16.35) | 385, 382, 367, 315, 289, 213 | C_28_H_48_O | Sterol | **Hannan et al., 2020** |
|  | Lobophytol A | 332.4 | 333.4  (24.87) | 331.4  (22.55) | 314 | C_20_H_28_O_4_ | Sterol | **Sohn et al., 2021** |
|  | sargachromenol | 424.6 | 425.6  (24.97) | - | 406, 360 | C_27_H_36_O_4_ | Terpenes | **Saraswati *et al*., 2019** |
|  | Chlorophyllide a | 614.2 | 615.2  (25.30) | - | 596, 546 | C_35_H_34_MgN_4_O_5_ | Pigment | **Dodds and Whiles, 2020** |
|  | fucosterol | 412.7 | 413.7 (25.48) | 411.7  (25.88) | 314, 298, 280, 228 | C_29_H_48_O | Sterol | **Ikekawa et al., 1968; Sohn et al., 2021** |
|  | Sargachromanol C | 412.7 | 413.7  ( 25.58) | 411.7  (25.95) | 394, 376 | [C_27_H_40_O_3_](https://pubchem.ncbi.nlm.nih.gov/#query=C27H40O3) | Terpenes | **Birringer et al.,2018** |
|  | Delphinidin chloride | 338.7 | 339.7  (25.60) | - | 320, 302 | C_15_H_11_ClO_7_ | Pigment | **Balasubramaniam et al., 2020** |
|  | Stypotriol triacetate | 554.7 | - | 553.7  (27.39) | 536, 518 | [C_27_H_40_O_4_](https://pubchem.ncbi.nlm.nih.gov/#query=C27H40O4) | Terpenes | **Kwon *et al*.,2022** |
|  | Chlorophyll d | 895 | - | 894  (28.89) | 877 | C_54_H_70_MgN_4_O_6_ | Pigment | **Cikoš et al., 2022** |
|  | 19-but-fucoxanthin | 744.9 | - | 743.9  (28.99) | 726, 708 | C_46_H_68_O_6_ | Pigment | **Pereira *et al*., 2021** |

Supplementary Table 7: LC/MS-MS of the pigment extract of *G. rugosa* using positive and negative ion acquisition modes.

| **No.** | **Tentative identification** | **M.wt** | **[M+H]^+^**  **(Rt)** | **[M-H]^-^**  **(Rt)** | **Characteristic fragments** | **Structure** | **Class** | **Ref.** |
| --- | --- | --- | --- | --- | --- | --- | --- | --- |
|  | Labdane | 278.5 | 279.5  (19.30) | 277.5  (6.93) | 180, 98 | [C_20_H_38_](https://pubchem.ncbi.nlm.nih.gov/#query=C20H38) | Terpenes | **Saraswati *et al*., 2019** |
|  | Cholesterol | 386.7 | 387.7  (24.49) | 385.7  (15.21) | 368[M-H_2_O], 160, 146, 108, 94 | [C_27_H_46_O](https://pubchem.ncbi.nlm.nih.gov/#query=C27H46O) | Sterol | **Ikekawa I., 1968; Sohn et al., 2021; Lopes et al., 2013** |
|  | Phytol | 296.5 | 297.5  (19.85) | 295.5  (15.42) | 281, 265, 253 | [C_20_H_40_O](https://pubchem.ncbi.nlm.nih.gov/#query=C20H40O) | Terpenes | **Rushdi et al., 2020** |
|  | β -carotene | 536.4 | - | 535.4  (15.71) | 444, 135, 429, 201,241,267,293 | C_40_H_56_ | Pigment | **Ibrahim *et al*., 2021a;Pereira *et al*., 2021** |
|  | Pelargonidin chloride | 306.7 | - | 305.7  (15.81) | 288, 270 | [C_15_H_11_ClO_5_](https://pubchem.ncbi.nlm.nih.gov/#query=C15H11ClO5) | Pigment | **Balasubramaniam *et al*., 2020** |
|  | Tuberatolide B | 422.6 | - | 421.6  (15.89) | 404, 358 | [C_27_H_34_O_4_](https://pubchem.ncbi.nlm.nih.gov/#query=C27H34O4) | Terpenes | **Saraswati *et al*., 2019; Kwon *et al*.,2022** |
|  | Erythrin | 422.4 | - | 421.4  (15.95) | 404, 386, 340 | C_20_H_22_O_10_ | Pigment | **Dodds and Whiles, 2020** |
|  | Lutein | 568.6 | - | 567.6  (16.23) | 549,475,428,411 | [C_40_H_56_O_2_](https://pubchem.ncbi.nlm.nih.gov/#query=C40H56O2) | Pigment | **Ibrahim *et al*., 2021a;Balasubramaniam *et al*., 2020** |
|  | Brassicasterol | 398.7 | - | 397.7  (16.28) | 380, 99 | C_28_H_46_O | Sterol | **Patterson,1971** |
|  | Hex-fucoxanthin, 19'-(SH) | 773 | - | 772  (16.35) | 755, 737, 673 | C_48_H_68_O_8_ | Pigment | **Pereira *et al*., 2021** |
|  | Campesterol | 400.7 | 401.7  (24.49) | 399.7  (16.39) | 385, 382, 367, 315, 289, 213 | C_28_H_48_O | Sterol | **Lopes et al., 2013** |
|  | cryptoxanthin | 552 | - | 551  (17.89) | 534, 496, 460 | C_40_H_56_O | Pigment | **Pereira *et al*., 2021** |
|  | (5E,9E,13E)-6,10,14-trimethyl pentadeca-5,9,13-trien-2,12-dione | 275 | - | 274  (17.97) | 219 | C_18_H_28_0_2_ | Terpenes | **Ryu et al., 2003** |
|  | Canthaxanthin | 564 | - | 563  (18.07) | 546, 458, 362, 202, 190, 132 | C_40_H_52_O_2_ | Pigment | **Pereira *et al*., 2021** |
|  | Cyclosadol | 440.7 | 441.7  (18.17) | - | 422 | [C_31_H_52_O](https://pubchem.ncbi.nlm.nih.gov/#query=C31H52O) | Terpenes | **Dembitsky *et al*., 2021** |
|  | Galaxaurol D | 471 | 472  (18.29) | - | 453, 435 | C_31_H_52_O_3_ | Terpenes | **Zhang et al.,2005** |
|  | Galaxaurol C | 467 | 468  (19.07) | - | 449, 387 | C_31_H_48_O_3_ | Terpenes | **Zhang et al.,2005** |
|  | Campestanol | 402.7 | 403.7  (18.39) | 401.7  (19.08) | 233, 219 | [C_28_H_50_O](https://pubchem.ncbi.nlm.nih.gov/#query=C28H50O) | Sterol | **Kircher and Rosenstein, 1974** |
|  | (5E,10Z)-6,10,14-trimethyl pentadeca-5,10-dien-2,12-dione | 277 | 278  (18.53) | 277.4  (19.30) | 221 | C_18_H_30_O_2_ | Terpenes | **Ryu et al., 2003** |
|  | Prostane | 280.5 | 281.5  (20.39) | - | 170, 76 | [C_20_H_40_](https://pubchem.ncbi.nlm.nih.gov/#query=C20H40) | Terpenes | **Kwon *et al*.,2022** |
|  | Ergosterol | 396.6 | - | 395.6  (22.25) | 312, 294, 270, 191, 153 | C_28_H_44_O | Sterol | **Lopes et al., 2013** |
|  | 22-dehydrocholesterol | 384.6 | 385.6  (24.49) | 383.6  (22.72) | 366, 158, 111, 69 | [C_27_H_44_O](https://pubchem.ncbi.nlm.nih.gov/#query=C27H44O) | Sterol | **Ikekawa I., 1968; Patterson,1971** |
|  | Pelargonidin | 271.2 | - | 270.2  (25.15) | 253, 235 | C_15_H_11_O_5_ | Pigment | **Haugan, 1994** |
|  | Stypotriol triacetate | 554.7 | - | 553.7  (25.24) | 536, 518 | [C_27_H_40_O_4_](https://pubchem.ncbi.nlm.nih.gov/#query=C27H40O4) | Terpenes | **Kwon *et al*.,2022** |
|  | Fucosterol | 412.7 | 413.7  (25.44) | - | 314, 298, 280, 228 | C_29_H_48_O | Sterol | **Sohn et al., 2021; Patterson,1971;** |
|  | 19-but-fucoxanthin | 744.9 | - | 743.9  (25.56) | 726, 708 | C_46_H_68_O_6_ | Pigment | **Pereira *et al*., 2021** |
|  | Adonirubin | 580.5 | 581.5  (20.39) | 579.5  (25.69) | 562, 91 | C_40_H_52_O_3_ | Pigment | **Pereira *et al*., 2021** |
|  | Cycloartenol | 426.7 | 427.7  (24.49) | 425.7  (25.71) | 408, 69 | [C_30_H_50_O](https://pubchem.ncbi.nlm.nih.gov/#query=C30H50O) | Sterol | **Dembitsky *et al*., 2021** |
|  | Cycloartane | 412.7 | 413.7  (25.54) | 411.7  ( 25.80) | 280 | [C_30_H_52_](https://pubchem.ncbi.nlm.nih.gov/#query=C30H52) | Terpenes | **Zhang et al.,2005** |
|  | Chlorophyll C2 | 608.2 | - | 607.2  (26.84) | 590, 544, 498 | [C_35_H_28_MgN_4_O_5_](https://pubchem.ncbi.nlm.nih.gov/#query=C35H28MgN4O5) | Pigment | **Cikoš et al., 2022** |
|  | Chlorophyll a | 893 | - | 892  (27.29) | 847 | [C_55_H_72_MgN_4_O_5_](https://pubchem.ncbi.nlm.nih.gov/#query=C55H72MgN4O5) | Pigment | **Cikoš et al., 2022** |
|  | Stigmasterol | 412.7 | - | 411.7  (28.05) | 254,158, 146, 96, 82, 68, 54 | [C_29_H_48_O](https://pubchem.ncbi.nlm.nih.gov/#query=C29H48O) | Sterol | **Patterson,1971** |
|  | Chlorophyll b | 907 | - | 906  (29.53) | 861, 831 | [C_55_H_70_MgN_4_O_6_](https://pubchem.ncbi.nlm.nih.gov/#query=C55H70MgN4O6) | Pigment | **Cikoš et al., 2022** |
|  | Delphinidin chloride | 338.7 | 339.7  (25.44) | 337.7  ( 30.19) | 320, 302 | C_15_H_11_ClO_7_ | Pigment | **Balasubramaniam et al., 2020** |
|  | Pheophytin a | 870.6 | - | 869.6  (30.40) | 592, 532, 459 | [C_55_H_74_N_4_O_5_](https://pubchem.ncbi.nlm.nih.gov/#query=C55H74N4O5) | Pigment | **Haugan, 1994** |
|  | Neoxanthin | 600 | - | 599  (30.54) | 582, 416, 296, 254 | [C_40_H_56_O_4_](https://pubchem.ncbi.nlm.nih.gov/#query=C40H56O4) | Pigment | **Balasubramaniam et al., 2020** |
|  | 23(E)-25-methoxy cycloart-23-en-3β-ol | 455 | - | 454  (31.47) | 437 | C_31_H_52_O_2_ | Terpenes | **Zhang et al.,2005** |

Supplementary Table 8: DPPH antioxidant activity of *S. aquifolium* and *G. rugosa.*

| Concentration | **% of antioxidant activity** | | | | | | |
| --- | --- | --- | --- | --- | --- | --- | --- |
|  | ***S. aquifolium* extracts** | | | ***G. rugosa* extracts** | | | **Ascorbic acid** |
|  | Pet.ether | Aqueous | Pigment | Pet.ether | Aqueous | Pigment |  |
| **10 µg** | 86.10 | 72.22 | 68.80 | 82.33 | 82.33 | 72.18 | 79.83 |
| **50** **µg** | 89.77 | 77.51 | 69.20 | 82.93 | 83.04 | 79.32 | 90.12 |

Supplementary Table 9: Cytotoxicity concentration 50 (CC50) and inhibitory concentration 50(IC50) of different extracts of *S. aquifolium* and *G. rugosa.*

| Macroalga | Pet.ether extracts | Aqueous extracts | Pigment extracts |
| --- | --- | --- | --- |
| *S. aquifolium* | 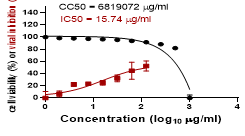 | 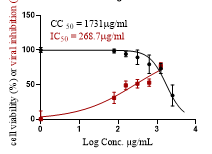 | 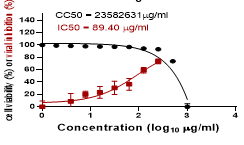 |
| *G. rugosa* | 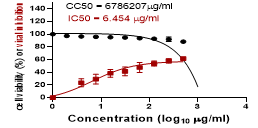 | 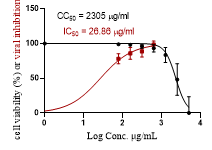 | 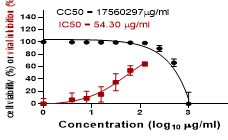 |

Supplementary Table 10: Cytotoxicity concentration 50 (CC_50_) and inhibitory concentration 50 (IC_50_) of different extracts of *s. aquifolium* and *g. rugosa* against SARS-COV-2

| **Macroalga** | **extract** | **CC_50_(µg/ml)** | **IC_50_(µg/ml)** |
| --- | --- | --- | --- |
| *S. aquifolium* | Pet.ether | 6819072 | 15.74 |
|  | Aqueous extract | 1731 | 268.70 |
|  | Pigment extract | 23582631 | 89.40 |
| *G. rugosa* | Pet.ether | 6786207 | 6.45 |
|  | Aqueous extract | 2305 | 26.86 |
|  | Pigment extract | 17560297 | 54.30 |
| Remdesivir | | 58.12 | 3.38 |

Supplementary Table 11: Cytotoxic activity of different macroalgal extracts against human breast cancer MCF7 cell line compared to doxorubicin.

| **Macroalga** | **extract** | **Cytotoxicity% at concentration (µg/ml)** | | | | | **IC_50_** |
| --- | --- | --- | --- | --- | --- | --- | --- |
|  |  | **6.25** | **12.5** | **25** | **50** | **100** |  |
| *S. aquifolium* | Pet.ether | 21.9 | 23.15 | 24.15 | 25.86 | 29.79 | 351.70 |
|  | Aqueous | 00 | 00 | 2.6 | 12.93 | 25.70 | 180.94 |
|  | Pigment | 24.15 | 26.20 | 30.30 | 38.44 | 44.20 | 114.49 |
| *G. rugosa* | Pet.ether | 4.77 | 9.8 | 11.23 | 13.95 | 21.80 | 260.83 |
|  | Aqueous | 13.78 | 14.80 | 17.01 | 25.35 | 29.60 | 196.01 |
|  | Pigment | 28.24 | 28.92 | 29.70 | 31.64 | 33.59 | 375.78 |
| Doxorubicin | | 29.96 | 41.85 | 60.23 | 81.74 | 93.01 | 25.70 |

IC_50_: Lethal concentration of the sample that causes the death of 50% of cells in 48 hours.

Supplementary Table 12: Cytotoxic activity of different macroalgal extracts against human lung cancer A549 cell line compared to doxorubicin.

| **Macroalga** | **extract** | **Cytotoxicity% at concentration (µg/ml)** | | | | | **IC_50_** |
| --- | --- | --- | --- | --- | --- | --- | --- |
|  |  | **6.25** | **12.5** | **25** | **50** | **100** |  |
| *S. aquifolium* | Pet.ether | 6.98 | 6.98 | 9.53 | 22.09 | 34.06 | 105.09 |
|  | Aqueous | 12.33 | 24.42 | 34.07 | 45.23 | 70.00 | 42.87 |
|  | Pigment | 8.14 | 20.77 | 28.20 | 38.84 | 62.56 | 51.42 |
| *G. rugosa* | Pet.ether | 6.98 | 9.07 | 21.19 | 27.19 | 44.03 | 75.64 |
|  | Aqueous | 8.02 | 18.84 | 33.24 | 43.95 | 55.12 | 51.47 |
|  | Pigment | 6.98 | 8.11 | 23.98 | 29.44 | 48.11 | 72.56 |
| Doxorubicin | | 32.33 | 38.87 | 59.77 | 76.63 | 90.96 | 26.85 |

Supplementary Table 13: Cytotoxic activity of different macroalgal extracts against human colon cancer HCT116 cell line compared to doxorubicin.

| **Macroalga** | **extract** | **Cytotoxicity% at concentration (µg/ml)** | | | | | **IC_50_** |
| --- | --- | --- | --- | --- | --- | --- | --- |
|  |  | **6.25** | **12.5** | **25** | **50** | **100** |  |
| *S. aquifolium* | Pet.ether | 6.98 | 6.98 | 9.53 | 16.12 | 25.68 | 123.05 |
|  | Aqueous | 16.39 | 30.93 | 42.56 | 53.72 | 76.02 | 36.19 |
|  | Pigment | 7.12 | 15.19 | 24.57 | 32.49 | 43.02 | 65.82 |
| *G. rugosa* | Pet.ether | 6.98 | 8.14 | 15.61 | 24.40 | 30.07 | 94.07 |
|  | Aqueous | 8.02 | 23.83 | 34.65 | 45.23 | 61.17 | 47.66 |
|  | Pigment | 6.98 | 6.98 | 18.84 | 26.34 | 38.87 | 84.85 |
| Doxorubicin | | 51.07 | 62.27 | 71.99 | 82.35 | 91.23 | 23.74 |

Supplementary Table 14: The calculated energy binding for the Candesalvone B and Salvimulticanol compounds against the catalytic binding site of IKKβ, and TLR4/MD-2 receptor

| **Energy Components (kcal/mol)** | | | | | |
| --- | --- | --- | --- | --- | --- |
| **main protease (Mpro) of SARS-CoV-2** | | | | | |
| **Complex** | **ΔE_vdW_** | **ΔE_elec_** | **ΔG_gas_** | **ΔG_solv_** | **ΔG_bind_** |
| **Phytol** | -37.64± 0.48 | -7.41± 0.60 | -45.05± 0.90 | 10.30± 0.39 | -34.75± 0.66 |
| **VEGFR2** | | | | | |
| **Phytol** | -54.70± 0.24 | -11.53± 0.21 | -66.24± 0.31 | 8.84± 0.22 | -57.39± 0.32 |

∆EvdW = van der Waals energy; ∆Eele = electrostatic energy; ∆Gsolv = solvation free energy; ∆Gbind = calculated total binding free energy.

Supplementary Table 15: Autodocking Vina docking results for isolated compounds docked into the catalytic domain binding site of Main protease Mpro in comparison to the co-crystallized ligand

| **Compounds** | **Hydrogen bonds between the atoms of compounds and the amino acids of the receptor** | | | | | **S-score (binding energy)(Kcal/mol)** |
| --- | --- | --- | --- | --- | --- | --- |
|  | Compounds | Receptor | | Type | Distance(A^0^) |  |
|  | atoms | atoms | residues |  |  |  |
| Cocrystalized ligand | H2521 | O2478 | Met162 | H-donor | 1.88 | -10.82 |
|  | O2513 | N4765 | PJE | H-acceptor | 3.07 |  |
| Hydroxycholestan-5-yl acetate | O4713 | OG690 | Ser46 | H-acceptor | 2.59 | -9.27 |
| Nonadecene | No interaction | | | | | |
| Phytol | O4703 | OG356 | Thr25 | H-donor | 2.29 | -10.67 |
|  | H4741 | O661 | Cys44 | H-donor | 1.83 |  |
|  | O4703 | OG356 | Thr25 | H-acceptor | 2.29 |  |
| Desmosterol | O4709 | OG342 | Thr24 | H-donor | 2.60 | -9.50 |
|  | O4709 | OG342 | Thr24 | H-donor | 2.60 |  |
| Nonadecanol | No interaction | | | | | |
| Cholesterol | O4710 | O367 | Thr26 | H-donor | 2.99 | -9.39 |
| 7-phenyl eicosane | No interaction | | | | | |
| 2-phenyl tridecane | No interaction | | | | | |
| Campesterol | No interaction | | | | | |
| 2-phenyl undecane | No interaction | | | | | |
| 4-phenyl dodecane | No interaction | | | | | |
| 6-phenyl dodecane | No interaction | | | | | |
| Tetracosene | No interaction | | | | | |

Supplementary Table 16: Autodocking vina docking results for isolated compounds docked into the catalytic domain binding site of VEGFR2 in comparison to the co-crystallized ligand.

| **Compounds** | **Hydrogen bonds between the atoms of compounds and the amino acids of the receptor** | | | | | **S-score (binding energy)(Kcal/mol)** |
| --- | --- | --- | --- | --- | --- | --- |
|  | Compounds | Receptor | | Type | Distance(A^0^) |  |
|  | atoms | atoms | residues |  |  |  |
| Cocrystalized ligand | H4945 | OE1230 | Glu885 | H-donor | 1.62 | -13.03 |
|  | H4932 | O1757 | Cys919 | H-donor | 2.08 |  |
| Hydroxycholestan-5-yl acetate | No interaction | | | | | |
| Nonadecene | No interaction | | | | | |
| Phytol | H4962 | O2579 | Ile1025 | H-donor | 2.96 | -11.28 |
|  | H4962 | O2598 | His1026 | H-donor | 2.37 |  |
|  | O4924 | NH2628 | Arg1027 | H-acceptor | 2.99 |  |
| Desmosterol | H4970 | O2599 | His1026 | H-donor | 3.16 | -9.45 |
|  | O4930 | NH2628 | Arg1027 | H-acceptor | 2.73 |  |
| Nonadecanol | No interaction | | | | | |
| Cholesterol | No interaction | | | | | |
| 7-Phenyl eicosane | No interaction | | | | | |
| 2-Phenyl tridecane | No interaction | | | | | |
| Campesterol | H44975 | O1164 | Ala881 | H-donor | 3.22 | -10.37 |
|  | O4931 | OG1214 | Ser884 | H-donor | 3.30 |  |
| 2-Phenyl undecane | No interaction | | | | | |
| 4-Phenyl dodecane | No interaction | | | | | |
| 6-Phenyl dodecane | No interaction | | | | | |
| Tetracosene | No interaction | | | | | |

| 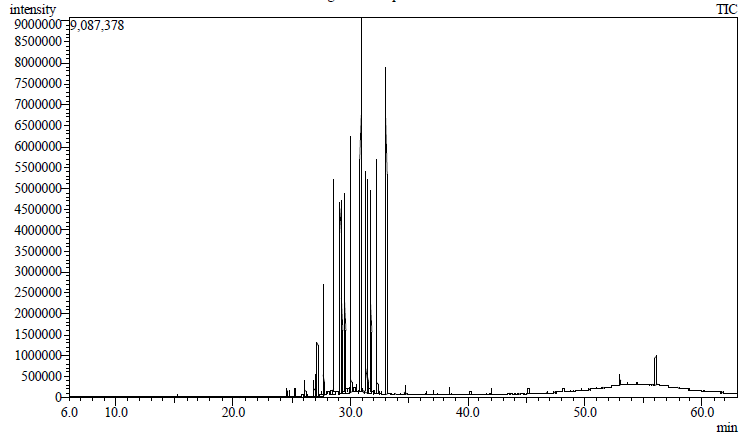  A |
| --- |
| **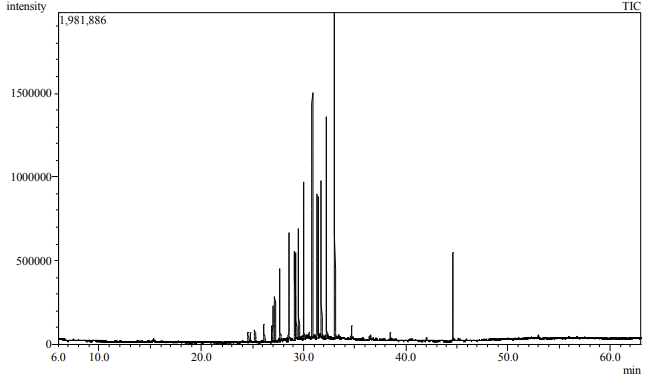**  B |

Supplementary Figure 1: GC/MS chromatogram of the USM of A: *S. aquifolium* and B: *G. rugosa*

| **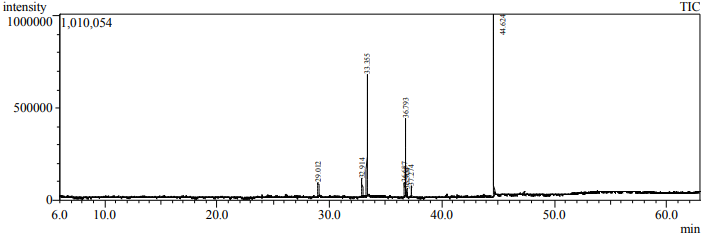**  A |
| --- |
| **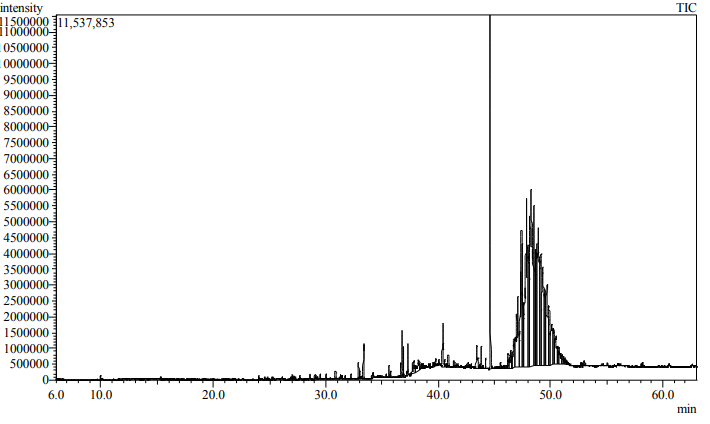**  B |

Supplementary Figure 2: GC/MS chromatogram of the FAME of A: *S. aquifolium* and B: *G. rugosa.*

| **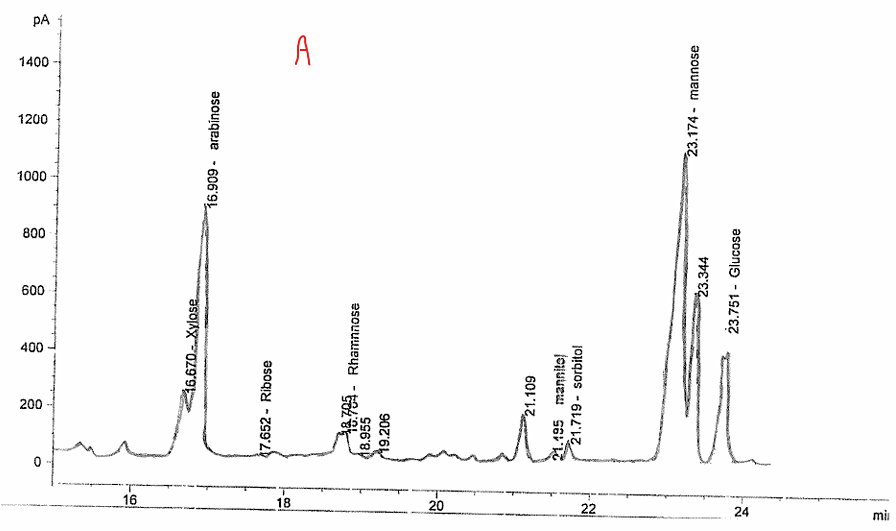**  A |
| --- |
| **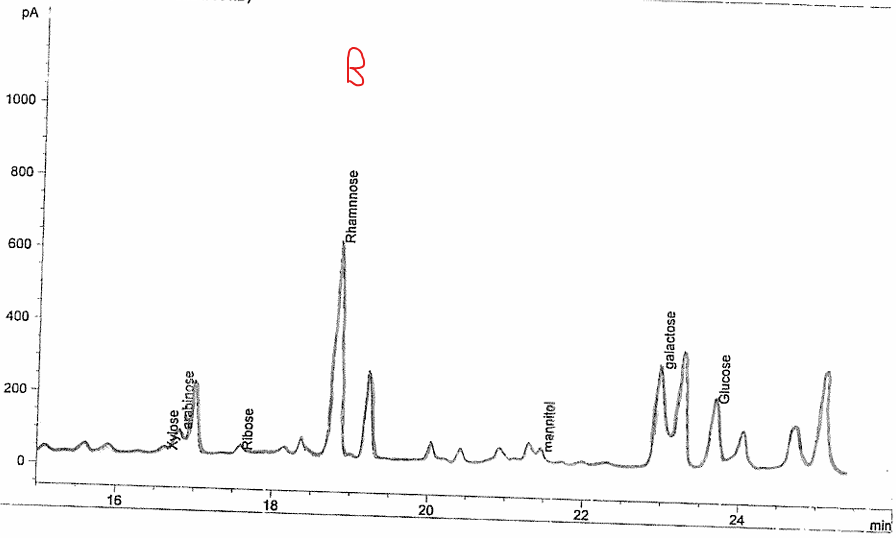**  B |

Supplementary Figure 3: GLC chromatogram of the monosugar content of the macroalgal isolated polysaccharides for *G. rugosa* (A) and for *S. aquifolium* (B).

| 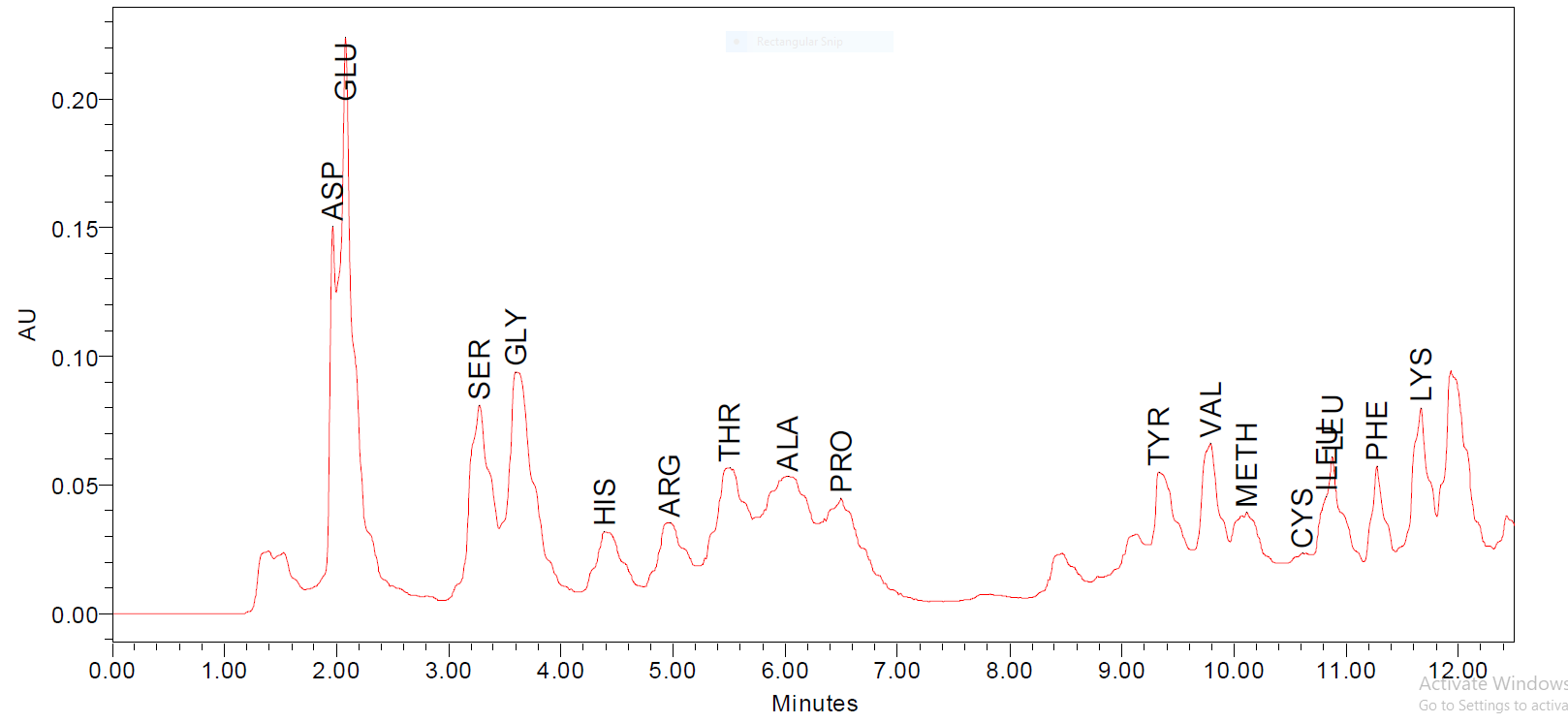  A |
| --- |
| 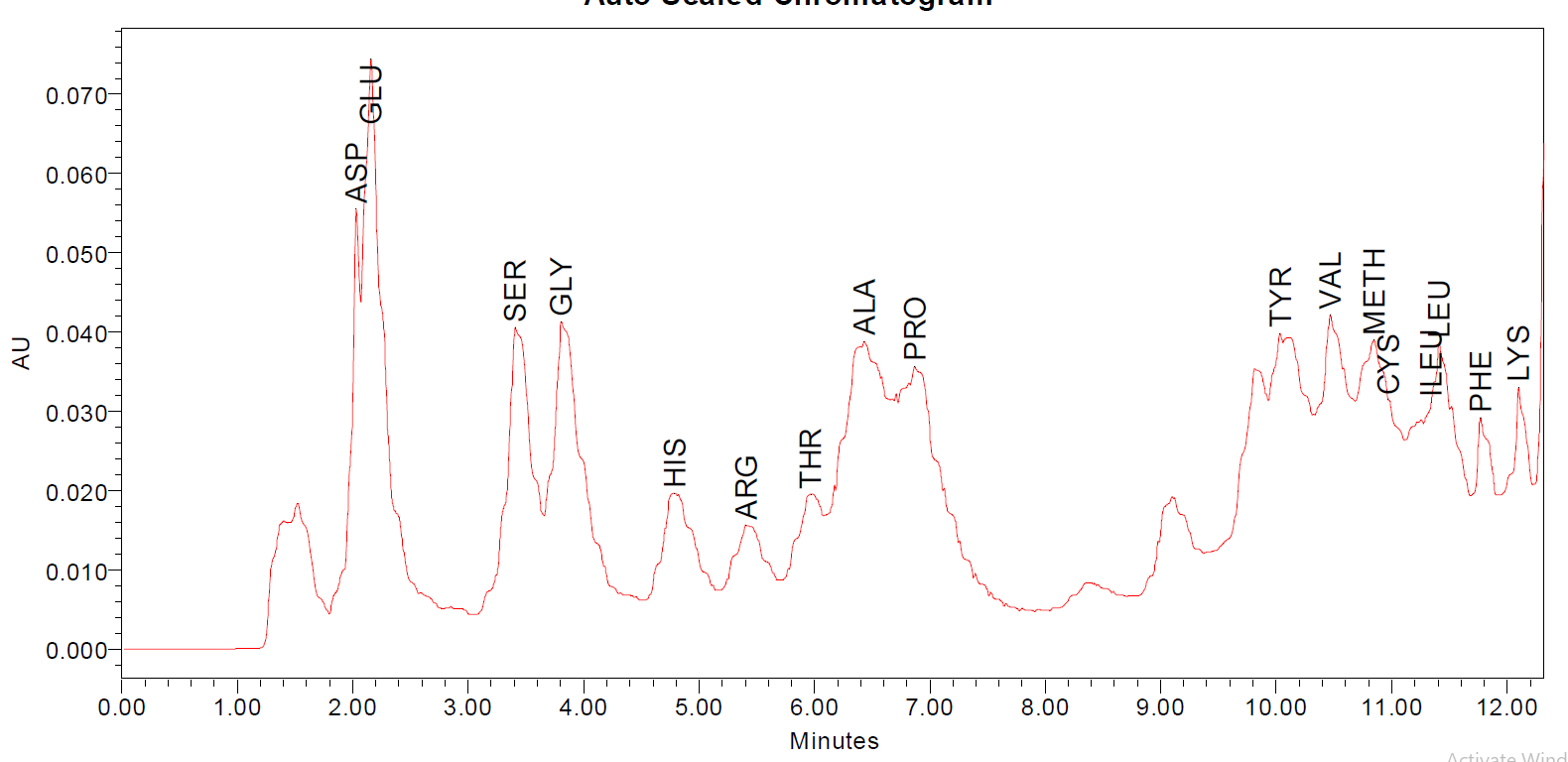  B |

Supplementary Figure 4: HPLC Chromatogram of proteins prepared from *S. aquifolium* (A) and *G. rugosa* (B).

| 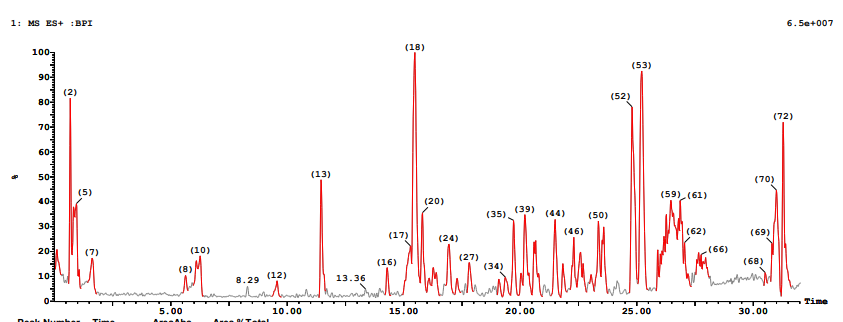  A |
| --- |
| 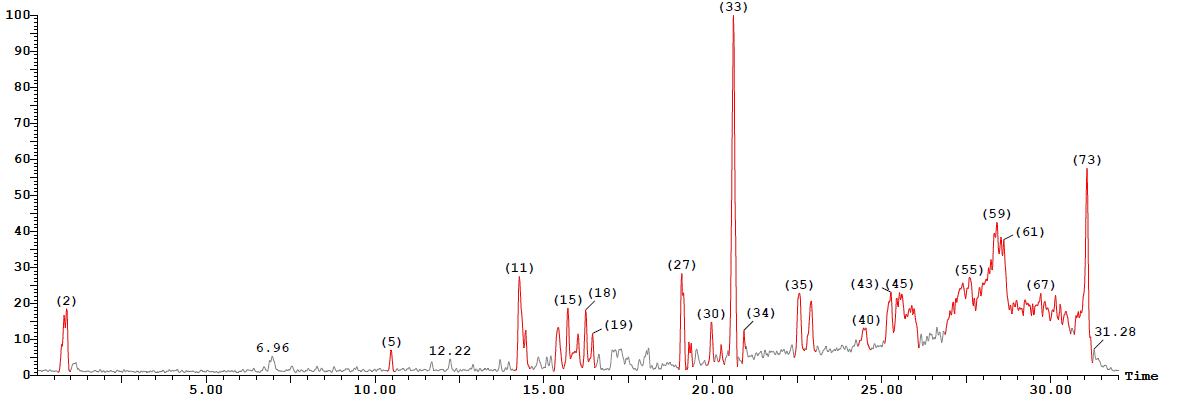  B |
| 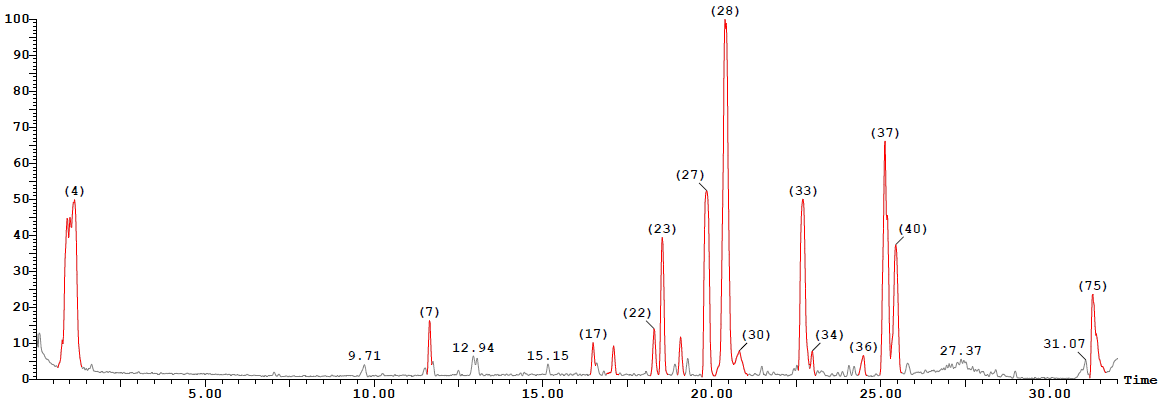  C |
| 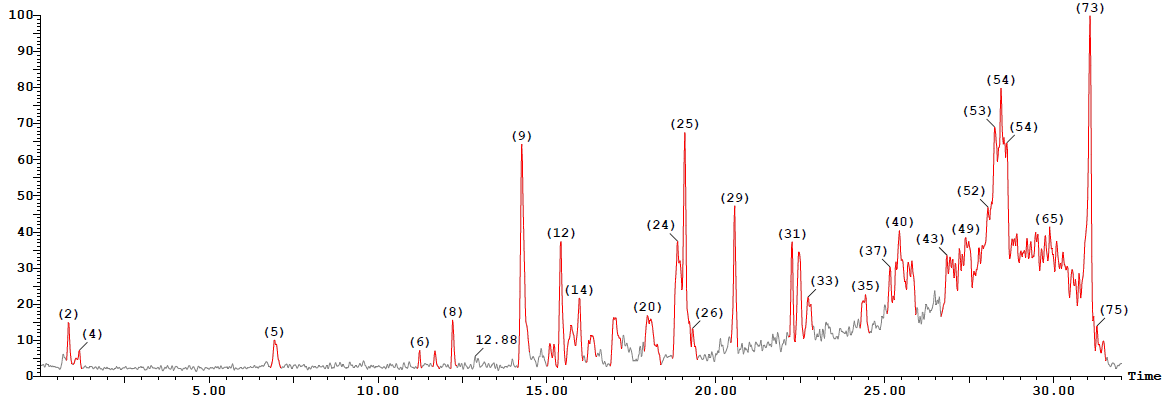  D |

Supplementary Figure 5: LC/MS-MS chromatograms of the pigment extracts of *S. aquifolium* and *G. rugosa* using positive (A, C) and negative (B,D) ion acquisition modes.


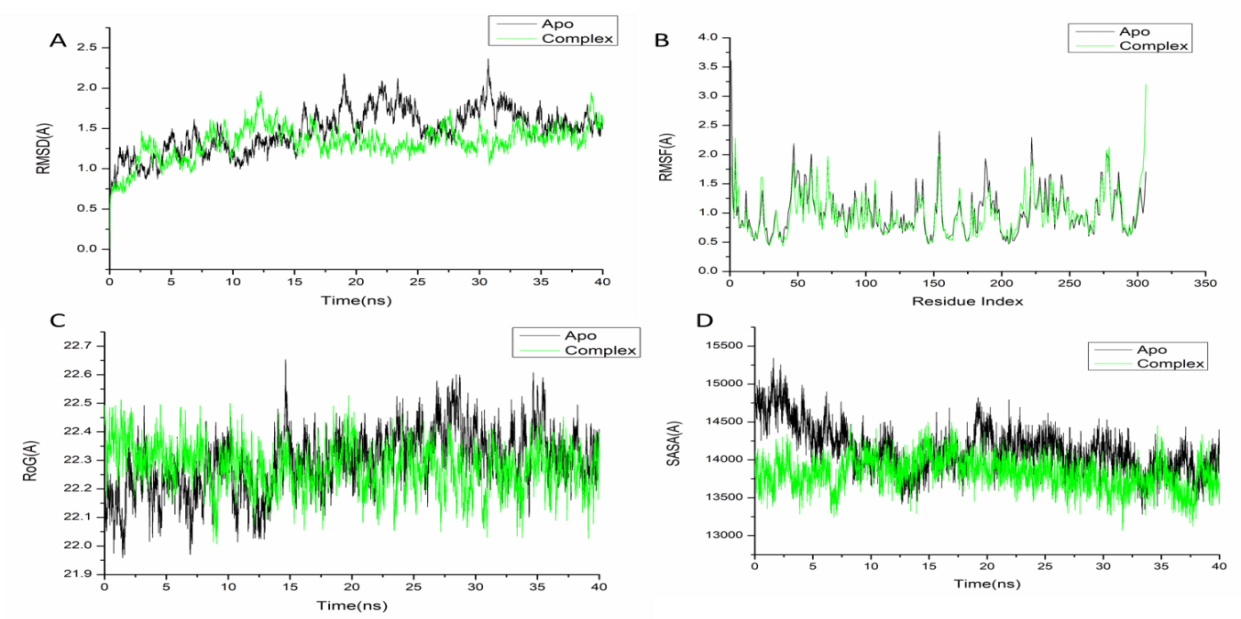


Supplementary Figure 6: [A] RMSD of Cα atoms of the protein backbone atoms. [B] RMSF of each residue of the protein backbone Cα atoms of protein residues (c) ROG of Cα atoms of protein residues; (d) solvent accessible surface area (SASA) of the Cα of the backbone atoms relative (blue) to the starting minimized over 40 ns for the ATP binding site of the main protease (Mpro) of SARS-CoV-2 receptor (black), and Phytol (green).


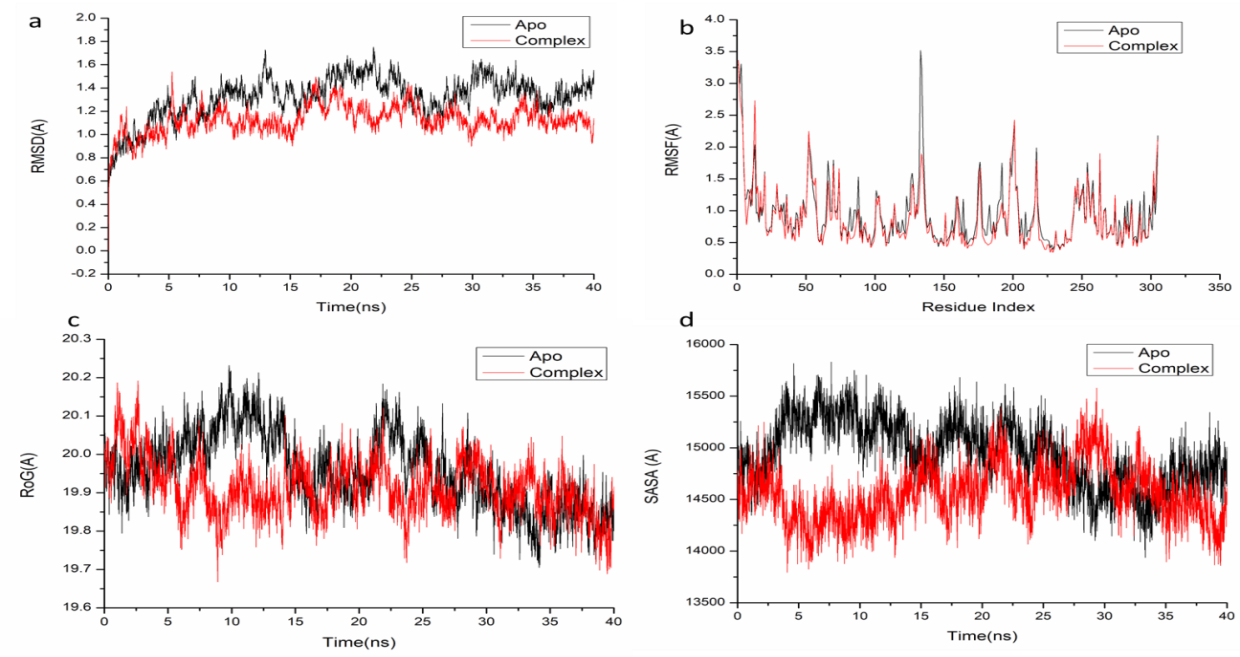


Supplementary Figure 7: [A] RMSD of Cα atoms of the protein backbone atoms. [B] RMSF of each residue of the protein backbone Cα atoms of protein residues (c) ROG of Cα atoms of protein residues; (d) solvent accessible surface area (SASA) of the C α of the backbone atoms relative (blue) to the starting minimized over 40 ns for the ATP binding site of VEGFR2 receptor (black),and Phytol (red).


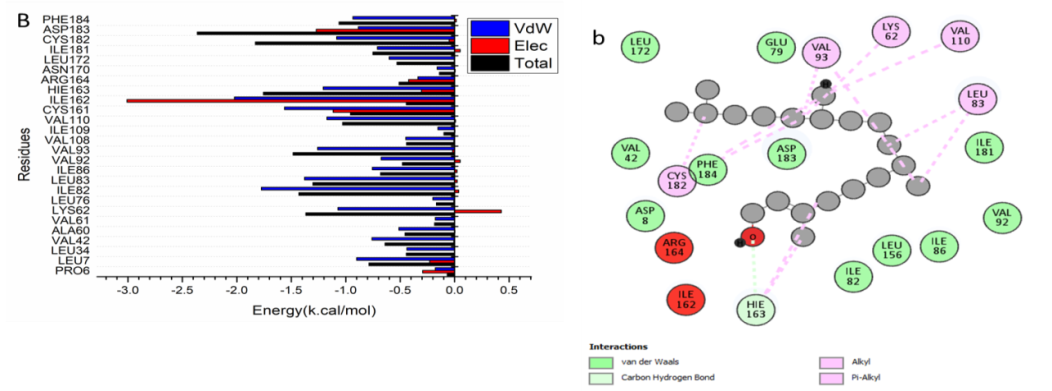

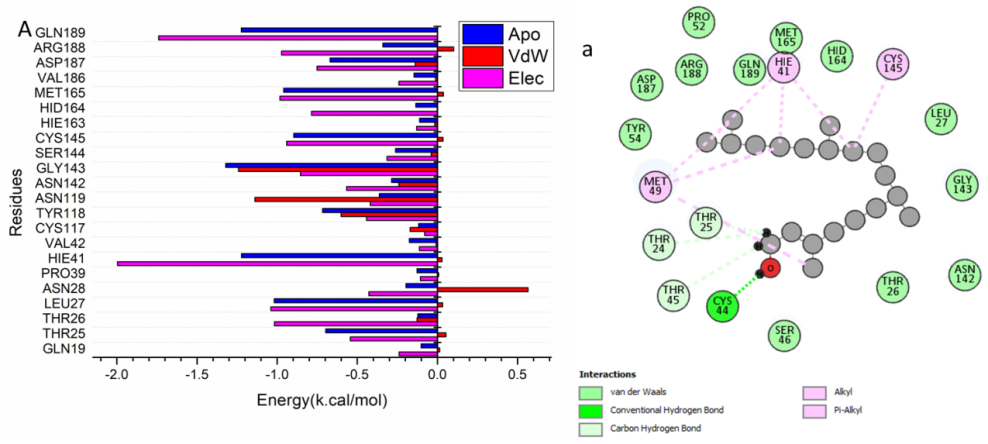


Supplementary Figure 8: Per-residue decomposition plots showing the energy contributions to the binding and stabilization of Phytol to the ATP binding site of Mpro [A] and VEGFR2 [B] receptor.


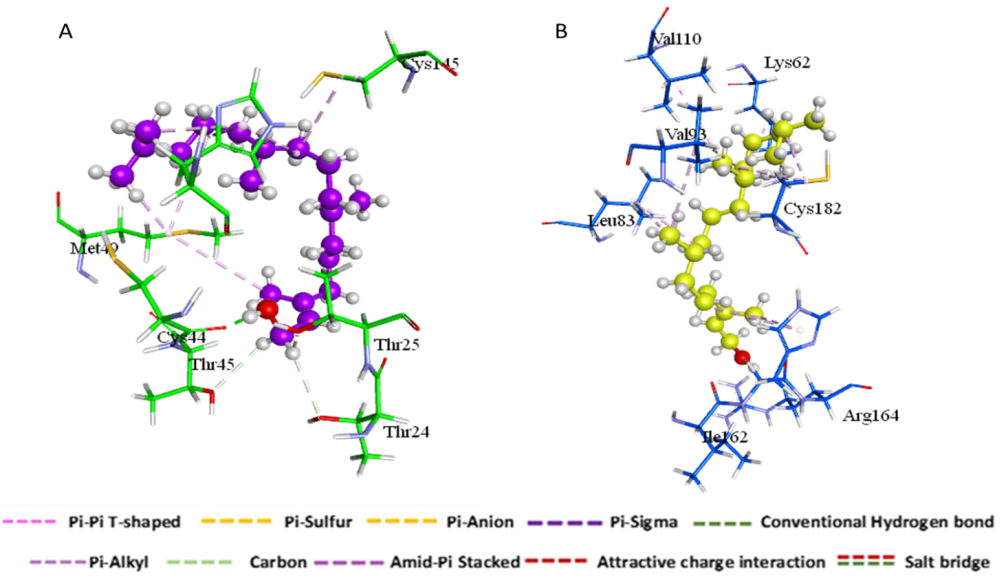


Supplementary Figure 9 : The phytol compound's contact residue in the Mpro [A] and VEGFR2 [B] receptor
